# Strategy to Evaluate Changes in Bacterial Community Profiles and Bacterial Pathogen Load Reduction After Sewage Disinfection

**DOI:** 10.3389/fmicb.2022.919207

**Published:** 2022-07-11

**Authors:** Mandy Lok Yi Tang, Stanley Chun Kwan Lau

**Affiliations:** Department of Ocean Science, Hong Kong University of Science and Technology, Hong Kong, China

**Keywords:** chlorination, effluent, pathogen, bacterial community, indicator bacteria, PMA treatment, quantitative PCR, amplicon sequencing

## Abstract

Sewage effluent discharge is a major source of pathogenic contamination to the environment. The disinfection process is critical for the elimination of pathogens in sewage. In this study, we examined the impact of chlorine disinfection on the total, viable, and culturable populations of indicator bacteria, pathogens, and bacterial communities in two contrasting types of effluents (primarily treated saline and secondarily treated freshwater). Effluents collected bimonthly over 1 year were examined using cultivation, quantitative PCR (qPCR), and 16S rRNA gene amplicon sequencing coupled with or without propidium monoazide (PMA) treatment. The results showed that each type of effluent was characterized by a specific set of representative genera before disinfection. Salinity appeared to be the major abiotic factor associated with the differences in bacterial community compositions. The pathogen analysis pipeline revealed over 20 viable clinically important pathogenic species in the effluents. Although the bacterial communities differed markedly between the two types of effluents before disinfection, the species of pathogens persisting after disinfection were similar, many of them were members of *Enterobacter* and *Vibrio*. The relative abundances of all pathogens identified in the amplicon sequences were multiplied by the 16S rRNA gene copy numbers of total bacteria detected by PMA-qPCR to estimate their concentrations. Pathogens remained viable after disinfection reached 8 log_10_ 16S rRNA copies ml^−1^ effluent. Meanwhile, around 80 % of the populations of three indicator bacteria including *Escherichia coli, Enterococcus*, and Bacteroidales were viable after disinfection, but over 99 % of the viable *E. coli* and *Enterococcus* were in the non-culturable state. We estimated the total pathogen load by adding the concentrations of all viable pathogens and examined their correlations with indicator bacteria of different types, physiological states, and effluents. The results showed that the PMA-qPCR measurement of *E. coli* is a reliable proxy of bacterial pathogen loads in both types of effluents. The utility of viable indicator bacteria as a biological index to assess the overall bacteriological hazards in effluents is discussed.

## Introduction

Sewage contains a large number of pathogens. To protect public health, effluents are often disinfected prior to the discharge to the receiving waters. The metabolism, physiology, and survival of bacteria in disinfected effluents have been an area of active research, but investigations have largely focused on indicator bacteria or a small number of pathogens that are readily culturable in the laboratories. These results are far from being representative of the vast majority of the non-culturable bacterial microbiome in sewage.

For decades, regulatory agencies rely on the culture-based enumerations of fecal indicator bacteria (FIB) (e.g., fecal coliforms, *Escherichia coli*, or *Enterococcus*) as the standard indicators of fecal pollution (USEPA, 2012). The reliability of bacterial cultivation for evaluating disinfection outcomes has been criticized in many reports (Schottroff et al., 2018; Momba et al., 2019; Di Cesare et al., 2020). First, the lack of colony growth does not imply the absence of viable cells. Disinfection processes, such as chlorination, can induce bacteria into the viable but non-culturable (VBNC) state (Oliver et al., 2005; Pienaar et al., 2016; Lin et al., 2017). VBNC cells are in a non-reproductive physiological state. Therefore, they lost the capability to form a colony on culture media. However, they are structurally and metabolically intact (Li et al., 2014a). VBNC state not only allows bacteria to survive prevailing environmental stressors (e.g., oxidative stress) but also prepares them with the ability to endure other harsh conditions, such as starvation, antibiotics, and extreme temperatures. After release from the stress factor, these bacteria are able to resuscitate in suitable hosts or environments (Oliver et al., 2005; Lin et al., 2017).

Second, the presence of indicator bacteria may not associate with the presence of a pathogen. Studies observed different survival rates and properties of indicators and pathogens (Harwood et al., 2005; Baker et al., 2021). However, due to low pathogen concentrations and difficulties in detection methods, studies are mainly based on pathogens that are readily culturable, isolated, or quantified. Korajkic et al. (2018) reviewed the studies on the relationships between indicators and pathogens in sewage effluents published over 40 years, and they indicated two main deficiencies in the studies: (1) almost half of the studies did not report statistical analysis on the relationships and (2) the rest only reported the statistical relationships with one or several pathogenic strains. Even though significant relationships were found between specific pairs of indicator and pathogen, the relationship could not be extrapolated to other pathogenic species for the robustness of that indicator bacteria or the particular detection method. It has not reached a consensus on which type and detection method of indicator bacteria is the best pathogen surrogate. Since there are various types of pathogens existing in the sewage, the ideal strategy to protect public health from the risk of sewage discharge is to establish a monitoring scheme with indicators that can associate with the presence or concentrations of all known pathogenic species.

Culture-independent methods such as quantitative polymerase chain reaction (qPCR) and amplicon sequencing are effective tools to examine the non-culturable portion of the bacterial communities in sewage. When used in conjunction with a DNA-binding dye, such as propidium monoazide (PMA), extracellular and non-viable cell DNA are covalently cross-linked by the dye and hindered from detection in the downstream PCR and sequencing processes, allowing the detection of the viable microbes in specific (Kibbee and Örmeci, 2017). With more studies unraveling the possible interferences and optimizing the procedures of PMA treatment, the use of PMA in complex environmental sample matrices, such as sewage, has become more common in the past decade (Li et al., 2014b; Eramo et al., 2019). Applications of PMA-coupled qPCR or sequencing have discovered a great variety of viable cells in disinfected effluents (Pang et al., 2016; Kibbee and Örmeci, 2017). This raises concern about the presence of resistant microbes in disinfected effluents, particularly pathogens.

In this study, we utilized cultivation, amplicon sequencing, and qPCR methods coupled with PMA treatment to examine the community compositions of the total, viable, and culturable bacteria including pathogens on the microbiome level. Due to the variance of total bacterial loads in different samples, the population dynamics of the pathogens detected in amplicon sequences were estimated by multiplying the percentages of relative abundances with the 16S rRNA gene copies of total bacteria measured in PMA-qPCR (Acharya et al., 2020; Jian et al., 2020; Tettamanti Boshier et al., 2020). The overall pathogen loads were compared with three types of indicator bacteria, including *Enterococcus, E. coli*, and Bacteroidales. *Enterococcus* and *E. coli* are FIBs commonly used around the world, while Bacteroidales is an emerging indicator for microbial source tracking due to its host-specific characteristics. Bacteroidales is more abundant than *E. coli* in fecal sources, its gene markers have been identified to differentiate fecal pollution from different sources (e.g., humans, cows, and pigs) (Silkie and Nelson, 2009; Liu et al., 2010). The values of Bacteroidales gene markers have been acknowledged by their comparable performance to the traditional FIBs (e.g., total coliforms, fecal coliforms, and *Enterococcus*) in predicting the presence of pathogens (Savichtcheva et al., 2007; Schriewer et al., 2010; Nshimyimana et al., 2017).

Two local sewage treatment facilities with contrasting types of effluents were involved in investigating the alterations and correlations of bacteria in different types of sewage before and after chlorine disinfection. One is a large-scale chemically enhanced primary treatment (CEPT) facility for treating saline influents, and another is a secondary treatment facility for freshwater influents. Saline sewage is treated because some coastal regions of Hong Kong use seawater for flushing. Nevertheless, the two treatment facilities receive only sewage without rainwater combined. Both facilities apply sodium hypochlorite solution for disinfection before the discharge of effluents. This study focused on several aspects of the bacteria in sewage under chlorine disinfection: (i) the changes in total, viable, and culturable populations of FIB and the amount of VBNC cells; (ii) the changes in the diversity and composition of the total and viable bacterial communities in two contrasting types of effluents; (iii) the viability and abundances of bacterial pathogens; and (iv) the correlations between the concentrations of FIB and pathogen loads. The ultimate goal of this study is to explore a suitable biological index for assessing the overall bacteriological threats in effluents.

## Materials and Methods

### Sources of Effluents

Effluents were collected from two municipal sewage treatment works equipped with chlorine disinfection, namely Stonecutters Island Sewage Treatment Works (SC) and Stanley Sewage Treatment Works (ST). SC is a CEPT facility for saline influents, whereas ST is a secondary treatment facility for freshwater influents. The operational parameters of each sewage treatment work and the physicochemical data measured in this study are summarized in Supplementary Table 1.

### Sampling Design

Bimonthly sampling over 1 year (August 2019 to June 2020) was conducted at SC and ST. At each sewage treatment work, triplicates of treated effluent samples (4 L each) were collected in 5-min intervals from a location immediately before the addition of sodium hypochlorite, another triplicate of samples (4 L each) was collected in 5-min intervals from a downstream location immediately after the addition of sodium thiosulphate for dechlorination. The collection of samples after chlorine disinfection lagged behind those before disinfection by 20 min, which was approximately the time required for the effluents to travel downstream through the chlorine mixing chamber. An excess of 0.1 M sodium thiosulphate was added to the samples to exhaust any chlorine that might remain after the dechlorination process. Field blanks of autoclaved Milli-Q water were prepared to check for contamination during the sampling. All samples were stored in autoclaved polypropylene (PP) bottles on the ice during the transport to the laboratory and started processing within 2 h. Each sample was split in three ways for (i) the measurement of physicochemical parameters, (ii) plate cultivation of *E. coli* and *Enterococcus*, and (iii) PMA treatment followed by DNA extraction for qPCR and 16S rRNA gene amplicon sequencing.

### Physicochemical Parameters

Temperature, salinity, dissolved oxygen (DO), pH, and turbidity of the samples were measured on-site immediately after collection by using a multiparameter water quality sonde (YSI, USA). In the laboratory, biological oxygen demand (BOD_5_), total suspended solids (TSS), total nitrogen (TN), ammonia (NH_3_), nitrite (NO${}_{2}^{-}$), nitrate (NO${}_{3}^{-}$), total phosphorous (TP), phosphate (PO${}_{4}^{3-}$), and silicate (SiO${}_{3}^{2-}$) were measured according to the APHA methods (APHA, 2005). The measurements of each sample were performed in triplicates and averaged.

### Enumeration of Culturable *E. coli* and *Enterococcus* spp.

The concentrations of culturable *E. coli* and *Enterococcus* spp. in each sample were determined by using the membrane filtration method (USEPA, 2000). Each sample was serially diluted (up to 10^7^ fold) with autoclaved phosphate-buffered saline (PBS). Each dilution was prepared in triplicates and filtered through 47 mm diameter, 0.45 μm pore-sized cellulose nitrate membranes. The membranes were then placed on CHROMagar^TM^ ECC agar for the enumeration of *E. coli* (Ho and Tam, 1997) and mEI agar for the enumeration of *Enterococcus* (USEPA, 2000). Blue colonies were counted after incubation in the dark for 24 h at 37°C for *E. coli* and 41°C for *Enterococcus*. The average concentrations of the triplicates of *E. coli* and *Enterococcus* were reported as numbers of colony forming units (CFU) per ml of effluents.

### PMA Treatment and DNA Extraction

Six portions of equal volume were taken from each sample (100 ml for SC samples and 300 ml for ST samples). The difference in the amount of samples used for SC and ST was due to lower bacterial concentrations in ST samples and higher levels of suspended solids in SC samples. For each portion, bacterial cells were centrifugated at 12,000 rpm for 10 min. After discarding the supernatant, the cells were vortexed in 10 ml of autoclaved PBS, then centrifugated again and resuspended in 496.5 μl of PBS. After that, the six portions were each spiked with 1 μl of salmon testes DNA (Sketa DNA) (Sigma-Aldrich) as control of PMA treatment efficiency. Three portions receiving the PMA treatment were added with 2.5 μl of PMA (Biotium, Inc.) to achieve a final concentration of 100 μM (Kibbee and Örmeci, 2017). The other three portions without PMA treatment were added with 2.5 μl of PBS instead. All samples were then vortexed for 5 s and incubated at 30 °C for 10 min in dark with shaking. After incubation, photoactivation of PMA was carried out by exposing the samples for 30 min to a 455–460 nm blue LED light source to reach the maximum absorption of PMA without sample overheating (Desneux et al., 2015). The samples without PMA were treated with the same incubation and photoactivation processes. After that, the three portions with or without PMA treatment of each sample were combined and centrifuged at 15,000 rpm for 5 min to obtain cell pellets for DNA extraction.

DNA was extracted using AllPrep® DNA/RNA Mini Kit (Qiagen) following the manufacturer's instructions. To quantify the loss of DNA during the extraction process, plasmid DNA of the cytochrome b gene of barnacle *Balanus amphitrite* was added to the lysates at a concentration of 4.32 × 10^6^ copies ul^−1^ as a control for extraction efficiency. The DNA plasmids were prepared following the procedures described in Liu et al. (2015).

### QPCR

Each DNA sample was tested with six sets of TaqMan probe-based qPCR assay for the quantitation of the gene copy numbers of total bacteria, Bacteroidales, *Enterococcus, E. coli*, salmon DNA (PMA control), and barnacle DNA (extraction control) (Table 1). Primer sets were selected due to their prevalent use in effluent studies, high coverage, and specificity of the bacterial targets as evaluated by the TestPrime tool of the SILVA rRNA database (https://www.arb-silva.de). The specificity of the primers was 100 % according to the database. The 23S rRNA genes assay of *E. coli* was used due to its high sensitivity which enables the detection of low concentrations of viable *E. coli* (Chern et al., 2011; Truchado et al., 2016), while the 23S rRNA genes assay of *Enterococcus* is a well-established assay commonly used in quantitative studies of sewage pollution with over 90 % coverage for the genus (USEPA, 2012). Since no probe sequence had been designed for the primer set of barnacle cytochrome b gene in previous studies, a probe was designed in this study based on the template sequences and annealing temperature of the primers by using the Probe Design Tool provided by Eurofins Genomics (https://eurofinsgenomics.eu/).

**Table 1:** Primers and probes for qPCR assays.

**Organism**	**Gene**	**Primer/probe**	**Sequence (5'-3')**	**Amplicon size**	**References**
Total bacteria	16S rRNA	Forward (331-F)	TCCTACGGGAGGCAGCAGT	466 bp	Nadkarni et al., 2002
		Reverse (797-R)	GGACTACCAGGGTATCTAATCCTGTT		
		Probe	CGTATTACCGCGGCTGCTGGCAC		
Bacteroidales	16S rRNA	Forward	GGGGTTCTGAGAGGAAGGT	129 bp	USEPA, 2010
		Reverse	CCGTCATCCTTCACGCTACT		
		Probe	CAATATTCCTCACTGCTGCCTCCCGTA		
*Enterococcus*	23S rRNA	Forward (ECST748F)	AGAAATTCCAAACGAACTTG	92 bp	USEPA, 2012
		Reverse (ENC854R)	CAGTGCTCTACCTCCATCATT		
		Probe (GPL813TQ)	TGGTTCTCTCCGAAATAGCTTTAGGGCTA		
*E. coli*	23S rRNA	Forward	GGTAGAGCACTGTTTTGGCA	88 bp	Sivaganesan et al., 2019 (USEPA Draft Method C)
		Reverse	TGTCTCCCGTGATAACTTTCTC		
		Probe	TCATCCCGACTTACCAACCCG		
Salmon testes (Sketa)	ITS region 2 of rRNA	Forward	GGTTTCCGCAGCTGGG	77 bp	USEPA, 2012
		Reverse	CCGAGCCGTCCTGGTC		
		Probe	AGTCGCAGGCGGCCACCGT		
*Balanus amphitrite* (Barnacle)	Cytochrome b	Forward	GGACACTGCATGCTAATGGA	144 bp	Bacchetti De Gregoris et al., 2009; Liu et al., 2015; this study
		Reverse	AGGCAGCAGCCATAGTCAAG		
		Probe	AAATTCCCCGCGAGACGTGAAGAT		

*Target organism, target gene, DNA sequences, amplicon size, and references of each set of primers and probe are shown*.

Each reaction mixture contained 5 μl AceQ U+ Universal Probe Master Mix V2 (Vazyme Biotech), 0.2 μM each primer, 0.1 μM TaqMan probe, 1-3 μl DNA sample, and topped up to 10 μl with nuclease-free water. The thermal cycle was 10 min at 95 °C, 45 cycles of 95 °C for 15 s, and 60 °C for 1 min. Standard curves were constructed using the plasmid DNA of each gene target following the procedures in Liu et al. (2015). To evaluate PCR inhibition, 10- and 100-fold dilutions of each sample were tested to determine the differences in the C_t_ values between dilutions. Differences <3.2 (equivalent to 91.9 % efficiency) were considered as amplification inhibition. The DNA samples of concern would be re-run at a higher level of dilution until the amplification efficiency was satisfied. All qPCR assays were done in triplicates and averaged. The gene marker concentrations detected in qPCR were corrected for DNA extraction efficiency and PMA treatment efficiency calculated as follows:


$$\begin{array}{l} Extraction\;efficiency\;\left(\%\right)\\ =\frac{Detected\;barnacle\;DNA\;copy\;}{Spiked\;barnacle\;DNA\;copy}\times \;100\;\%\\ PMA\;efficiency\;\left(\%\right)\\ =\left(1-\frac{Detected\;salmon\;DNA\;copy\;}{Spiked\;salmon\;DNA\;copy}\right)\times \;100\;\% \end{array}$$

When comparing the concentrations of viable and culturable cells, the corrected copy numbers of the 23S rRNA genes of *E. coli* and *Enterococcus* were converted to cell number equivalents. According to curated rRNA gene copy numbers in Ribosomal RNA Database (rrnDB) (Stoddard et al., 2015), the median rRNA copy number per cell was 7 in *E. coli* and 6 in *Enterococcus*. The calculations of *E. coli* and *Enterococcus* cell equivalents were.


$$\begin{array}{l} Number\;of\;cell\;equivalent\\ =\frac{\;Gene\;copy\;number\;detected\;in\;qPCR}{Median\;of\;gene\;copy\;number\;per\;cell} \end{array}$$

### Amplicon Sequencing and Data Processing

The V3-V4 regions of the bacterial 16S rRNA genes in DNA samples were amplified using the primers 341F (5'- CCTAYGGGRBGCASCAG – 3') and 806R (5' -GGACTACNNGGGTATCTAAT – 3') with barcodes. The 466 bp amplicons were sequenced on an Illumina platform to generate 250 bp paired-end raw reads. The barcode and primer sequences were trimmed by using QIIME 2 2017.4 (Bolyen et al., 2019). The merging, quality filtering, denoising, and chimera removal of the paired-end reads were done on DADA2 using the default settings (Callahan et al., 2016). Taxonomic classification of the filtered reads was performed using the classify-sklearn method of the q2-feature-classifier plugin provided in QIIME 2 with the classifier trained on Silva v132 99 % identity clustered reference database (https://www.arb-silva.de/download/archive/).

For the identification of bacterial pathogens in the samples, raw reads were filtered and aligned against a 16S pathogen database covering 346 pathogenic species of human health concern using the online fast mode of the 16S Pathogenic Identification Process (16sPIP) analysis pipeline (Miao et al., 2017) (https://16spip.mypathogen.cn). Filtered reads with > 99 % similarity to the sequences in the database were mapped. Pathogenic species with relative abundances of < 0.01 % in the whole bacterial communities were excluded from downstream analysis. To estimate the concentrations of pathogens in each sample, the relative abundances of pathogens (the number of annotated pathogen reads over the number of total annotated bacterial reads) were multiplied by the copy numbers of total bacterial 16S rRNA genes determined in qPCR (Acharya et al., 2020; Jian et al., 2020; Tettamanti Boshier et al., 2020).

### Statistics Analysis and Data Visualization

The sequence variants (unique sequences distinguished by a single nucleotide change) after rarefying the filtered reads to the minimum depth (34,683 reads) were utilized for the alpha-diversity analysis and beta-diversity metrics. Kruskal–Wallis analysis was used to test for the differences between total and viable populations before and after chlorine disinfection in the alpha-diversity indices.

To determine whether a linear or unimodal model should be used in constrained ordination analysis, detrended correspondence analysis (DCA) was conducted using the vegan package in R software version 3.6.3 (https://www.r-project.org/). Since the calculated length of the first axis in DCA ordination exceeded 4 standard deviation (S.D.), a constrained correspondence analysis (CCA) plot was utilized as a unimodal model and constructed using vegan and ggplot packages in R. Permutational Multivariate Analysis of Variance (PERMANOVA) tests were performed for comparing the bacterial community similarity between sewage treatment works and identifying significant explanatory variables in CCA.

The visualization of taxa plots and Linear Discriminant Analysis Effect Size (LEfSe) test were supported by MicrobiomeAnalyst (https://www.microbiomeanalyst.ca/). Only the taxa with a minimum of 4 read counts in at least 20 % of all samples were retained for LefSe analysis.

The differences in the relative abundances and estimated concentrations of individual taxa, as well as the total, viable, and culturable concentrations of individual indicator bacteria before and after chlorine disinfection, were tested by paired *t*-test. The correlations between the concentrations of pathogens and fecal indicators were determined using Pearson's Correlation Coefficient and visualized in scatter plots using the ggscatter package in R.

## Results

### Total, Viable and Culturable Concentrations of Indicator Bacteria

**Figure 1:**
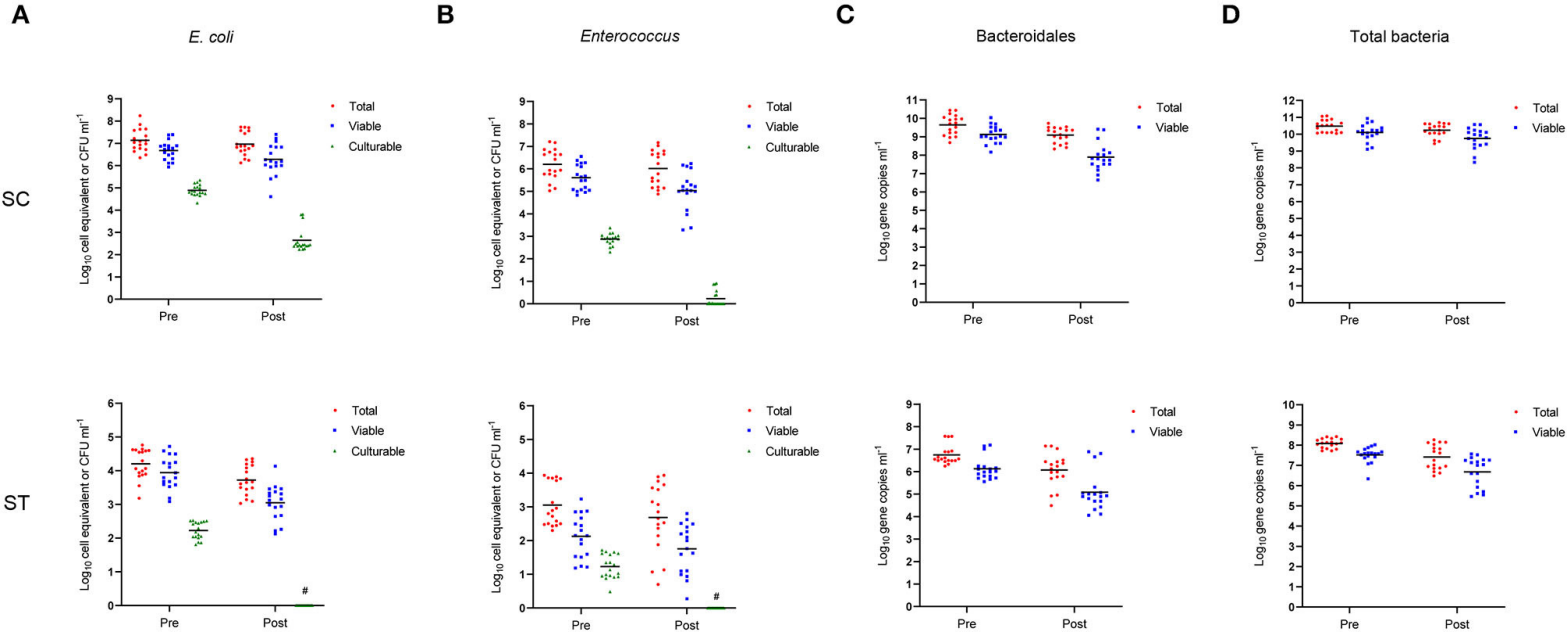
Concentrations of *E. coli, Enterococcus*, Bacteroidales, and total bacteria before (Pre) and after (Post) chlorine disinfection in SC and ST. The samples collected bimonthly over a year were represented by individual data points, and the arithmetic means of the samples were indicated by horizontal lines. The concentrations of total, viable and culturable cells of **(A)** *E. coli* and **(B)** *Enterococcus* in each sewage treatment work were compared. The total and viable 23S rRNA gene copy numbers detected in PMA-qPCR assays were converted to numbers of cell equivalent by dividing the median numbers of gene copies per cell (log_10_ cell equivalent ml^−1^). The concentrations of culturable cells were determined using the plate counting method (log_10_ CFU ml^−1^). The concentrations of total and viable 16S rRNA gene copy numbers of **(C)** Bacteroidales and **(D)** total bacteria in each sewage treatment work (log_10_ gene copies ml^−1^) were shown. # Less than 1 colony was found per ml of effluent.

The average culturable counts of *E. coli* and *Enterococcus* in the effluents of SC and ST were significantly reduced after chlorine disinfection over the six bimonthly sampling period (*p* < 0.001, paired *t*-test) (Figures 1A,B). The average culturable counts of *E. coli* over the year reduced from 4.89 ± 0.25 (mean ± 1 S.D.) to 2.64 ± 0.52 log_10_ CFU ml^−1^ in SC and from 2.23 ± 0.25 log_10_ CFU ml^−1^ to < 1 CFU ml^−1^ in ST, while that of *Enterococcus* reduced from 2.88 ± 0.25 to 0.23 ± 0.34 log_10_ CFU ml^−1^ in SC and from 1.23 ± 0.35 log_10_ CFU ml^−1^ to < 1 CFU ml^−1^ in ST. In general, chlorine disinfection in SC achieved over 2 log_10_ reduction in the culturable counts of *E. coli* and *Enterococcus*, while that in ST reduced both types of culturable cells below the detection limit (0.01 CFU ml^−1^).

For the culture-independent enumeration using qPCR with PMA treatment, the DNA extraction and PMA treatment efficiencies evaluated by barnacle and salmon testes DNA controls are listed in Supplementary Table 2. The extraction efficiencies of all SC and ST samples ranged from 0.06 to 9.54 %. Samples from ST had slightly higher average extraction efficiency (2.57 ± 2.06 %) than that of SC (1.41 ± 1.35 %). The average PMA treatment efficiency in ST samples (99.74 ± 0.41 %) was also higher than that of SC samples (79.89 ± 11.23 %). It was observed that the PMA treatment achieved consistently high efficiencies in all ST samples (> 97 %), while a wider range of efficiencies was observed among SC samples (54.36–93.63 %). The differences in DNA extraction and PMA treatment efficiencies between samples before and after chlorination were below 1 %. The variances in DNA extraction and PMA treatment efficiencies exhibited different strengths of correlations with the measured physicochemical parameters. Among all the physicochemical parameters, salinity, TN, and TP were the strongest factors negatively correlated to the extraction efficiencies of all samples (Kendall's Tau < −0.33, *p* < 0.001), while TSS and TN were the strongest factors negatively correlated to the PMA treatment efficiencies of all samples (Kendall's Tau < −0.59, *p* < 0.001). In addition, amplification inhibition of qPCR was observed in some of the SC and ST samples, up to 10× dilution was required to eliminate the inhibition (Supplementary Table 2).

In contrast to the culturable counts, the average viable concentrations of *E. coli* determined by the corrected PMA-qPCR gene copies over the year reduced by only 0.39 ± 0.33 log_10_ copies ml^−1^ in SC and 0.89 ± 0.44 log_10_ copies ml^−1^ in ST, while that of *Enterococcus* reduced by only 0.58 ± 0.48 log_10_ copies ml^−1^ in SC and 0.37 ± 0.21 log_10_ copies ml^−1^ in ST. The magnitudes of reduction in the total concentrations of *E. coli* and *Enterococcus* (including viable and non-viable cells) were lower, as indicated in the results of qPCR without PMA treatment. The average total concentrations of *E. coli* over the year reduced by 0.17 ± 0.10 log_10_ copies ml^−1^ in SC and 0.48 ± 0.40 log_10_ copies ml^−1^ in ST, while that of *Enterococcus* reduced by 0.19 ± 0.18 log_10_ copies ml^−1^ in SC and 0.36 ± 0.53 log_10_ copies ml^−1^ in ST.

Before disinfection, the average viable cell equivalents of *E. coli* and *Enterococcus* in SC were 6.67 ± 0.41 and 5.61 ± 0.55 log_10_ ml^−1^, respectively, while that in ST were 3.94 ± 0.46 and 2.12 ± 0.62 log_10_ ml^−1^, respectively (Figures 1A,B). After disinfection, the average viable cell equivalents of *E. coli* and *Enterococcus* in SC reduced to 6.28 ± 0.68 and 5.03 ± 0.85 log_10_ ml^−1^, respectively. The difference between the concentrations of viable and culturable cells of *E. coli* was 6.28 ± 0.69 log_10_ ml^−1^, while that of *Enterococcus* was 5.03 ± 0.85 log_10_ ml^−1^, both accounted for over 99 % of the viable populations. For ST, the average viable cell equivalents of *E. coli* and *Enterococcus* in the disinfected effluents were 3.05 ± 0.50 and 1.75 ± 0.72 log_10_ ml^−1^, respectively, but no culturable cells were detected after disinfection.

As an alternative fecal indicator, the total 16S rRNA gene copy numbers of Bacteroidales on average over the year reduced by 0.56 ± 0.19 log_10_ copies ml^−1^ in SC and 0.67 ± 0.51 log_10_ copies ml^−1^ in ST after disinfection, while the average viable copy numbers reduced by 1.29 ± 0.53 log_10_ copies ml^−1^ in SC and 1.04 ± 0.36 log_10_ copies ml^−1^ in ST (Figure 1C). Among the three fecal indicators (*E. coli, Enterococcus*, and Bacteroidales), Bacteroidales had the greatest reduction in the viable population. Besides, the average total and viable copy numbers of the 16S rRNA gene of total bacteria in SC reduced by 0.25 ± 0.14 and 0.35 ± 0.19 log_10_ copies ml^−1^, respectively, while that in ST reduced by 0.68 ± 0.43 and 0.83 ± 0.49 log_10_ copies ml^−1^, respectively (Figure 1D). The changes in the total and viable concentrations of the three indicator bacteria and total bacteria load in SC or ST did not show a distinct seasonal pattern (Supplementary Figure 1). One possible reason for the lack of an apparent seasonal pattern is the separation of storm water drainage from sewage drainage in Hong Kong, reducing the effects of seasonal terrestrial runoff on the bacterial concentrations in the influents.

Although chlorine disinfection reduced the viable concentrations of all four bacterial targets in both SC and ST, the viable concentrations of these bacteria occupied an average of 86.68 ± 10.26 % of their total concentrations in the effluents after disinfection, indicating that the majority of the bacterial populations in the disinfected effluents pending discharge were still alive, despite the significant disinfection efficacy manifested in the culturable counts of *E. coli* and *Enterococcus*.

### Bacterial Community Diversity in Two Contrasting Types of Effluents

The amplicon sequencing of 144 samples (two sewage treatment works, six bimonthly sampling, and triplicate samples before and after chlorine disinfection, with and without PMA treatment) resulted in an average of 67,310 ± 12,408 reads per sample after quality filtering. All samples had > 85 % of bases with quality scores over 30 (Q30). A total of 19,654 sequence variants were observed among all the samples. The alpha rarefaction curve showed that the observed sequence variants had reached a plateau at current sequencing depths (Supplementary Figure 2). The details of the sequencing data including raw and filtered read counts, Q30, and GC content of each sample are listed in Supplementary Table 3.

Bacterial community structures of the samples in two sewage treatment works, particularly the viable populations, are segregated along the first axis of the Principal Coordinates Analysis (PCoA) plot (Supplementary Figure 3). The first axis accounted for 57.44 % of the variation within the weighted UniFrac distance matrix of all samples. The variances between SC and ST samples in both total and viable populations were supported by PERMANOVA (*q* = 0.001, pseudo-F = 189, permutation = 999). The bacterial community structures of SC and ST were correlated with the physicochemical paraments of the effluents (Supplementary Figure 4). All the constraining variables were statistically significant (*p* < 0.05, PERMANOVA) except TSS. The bacterial community structures in SC had the strongest positive correlation with salinity, followed by turbidity, BOD_5_, TN, and NH_3_. In contrast, bacterial community structures in ST had negative correlations with these parameters but positive correlations with DO and NO${}_{3}^{-}$.

**Figure 2:**
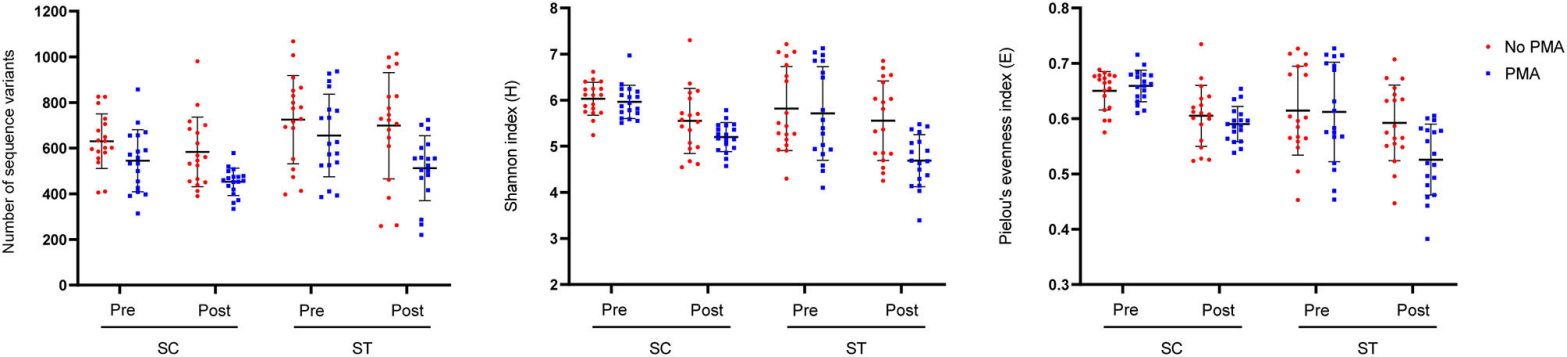
Alpha-diversity indices (number of sequence variants, Shannon index and Pielou's evenness index) measuring the richness, diversity, and evenness of total (No PMA) and viable (PMA) bacterial communities before and after chlorine disinfection. The samples collected bimonthly over a year were represented by individual data points with error bars (mean ± 1 S.D). All samples were rarefied to the minimum depth of filtered reads and the sequence variants were used for calculation.

In respect of alpha-diversity, the richness, diversity, and evenness of the viable bacterial communities were significantly reduced after chlorine disinfection in both SC and ST (*p* < 0.05, Kruskal-Wallis test) (Figure 2). In SC, the number of sequence variants of the viable populations reduced from 545 ± 132 to 452 ± 58.30 on average over the year. The reductions in Shannon's H and Pielou's E values after chlorine disinfection were both significant (*p* < 0.001). Similar results were found in the samples of ST, all the alpha-diversity indices of the viable populations decreased significantly after chlorine disinfection (*p* < 0.05). The average number of sequence variants in the viable populations of ST was reduced from 656 ± 176 to 512 ± 139 after disinfection. However, the alpha-diversity indices of the total populations in SC and ST did not change significantly (*p* > 0.05).

### Alterations in Taxonomic Compositions and Removal of Pathogens After Chlorine Disinfection

A total of 3,161 taxa corresponding to 58 phyla, 156 classes, 406 orders, 796 families, and 1,780 genera were identified in all samples of SC and ST. Among the 15 most abundant genera (covering over 83 % of all assigned reads), 12 of them belonged to Proteobacteria, and the rest was Bacteroidetes (Supplementary Figure 5). SC and ST had remarkable differences in the taxonomic compositions regardless of the chlorine disinfection. *Arcobacter* was the most dominant genus in SC throughout the year. It accounted for an average of 53.86 ± 9.45 % of all taxa in SC samples. In contrast, the dominant genera of ST varied between months, including *Aeromonas* (2.68–34.76 %), *Acinetobacter* (0.75–21.83 %), and *Vibrio* (0.57–60.75 %), each with a wide range of relative abundances in the ST samples across the year. The taxonomic profiles of ST showed higher variations over time than that of SC, reiterating the temporal community structure dynamics observed in the CCA plot.

**Figure 3:**
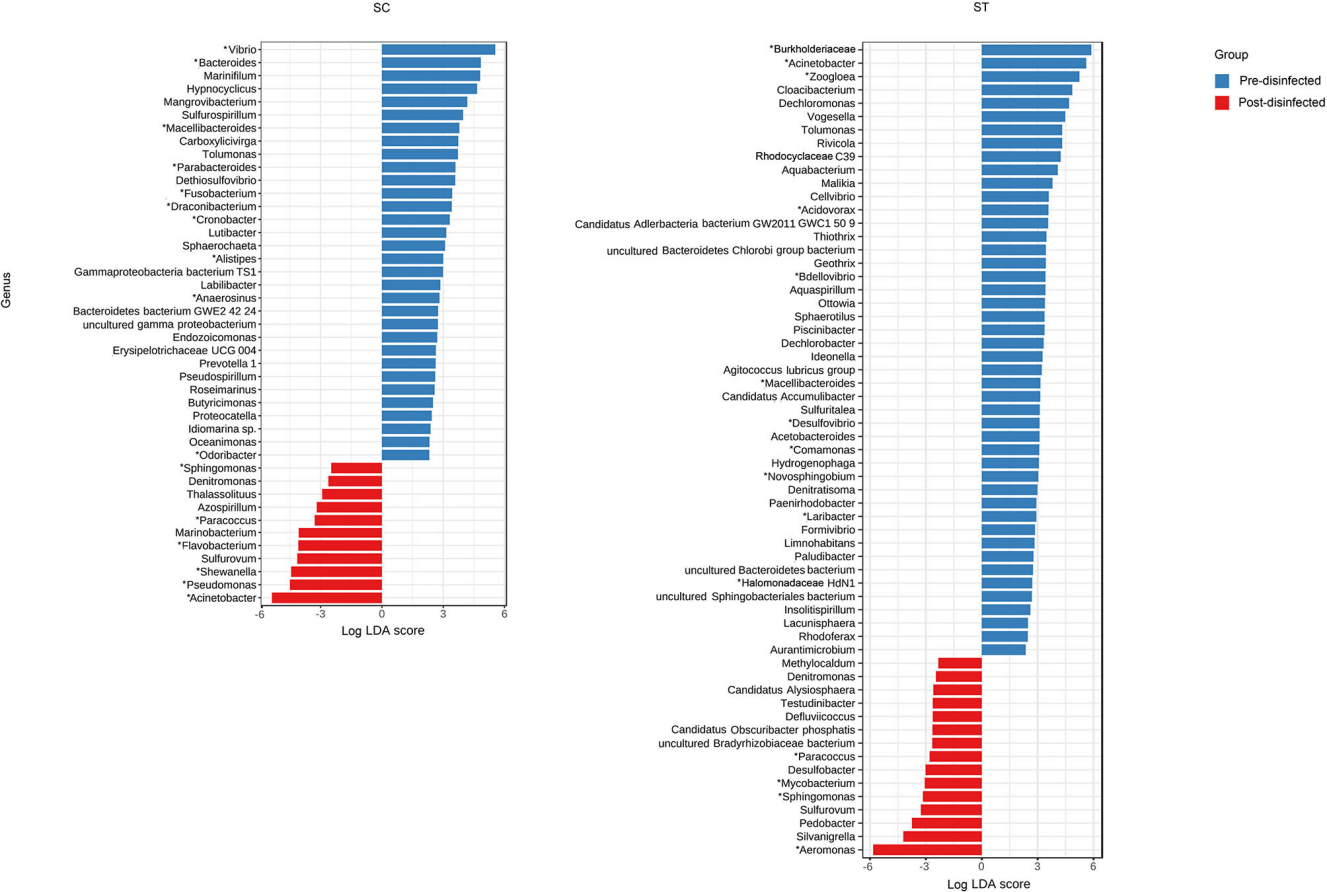
Linear Discriminant Analysis Effect Size (LEfSe) plot of significantly discriminative genera identified in the viable populations of effluents in SC and ST (Kruskal–Wallis sum-rank test, *q* < 0.05, absolute log LDA score > 2). Absolute LDA score indicates the discriminatory power of the genus in the dedicated group. Genera with reported pathogenic species were marked with asterisks.

Taxa that were most representative of the taxonomic compositions of the viable populations in the effluents before and after chlorine disinfection were determined by using the LEfSe analysis (Figure 3). It revealed 32 and 11 discriminative genera in the pre- and post-disinfected effluents of SC, respectively, and 46 and 15 genera in the pre- and post-disinfected effluents of ST, respectively (Bonferroni-adjusted *p*-value < 0.05, absolute log LDA score > 2, Kruskal–Wallis sum-rank test). The most representative genus in the pre-disinfected effluents of SC and ST were *Vibrio* and *Burkholderiaceae*, which exhibited significant reductions in relative abundances after chlorine disinfection (*p* < 0.05, paired *t*-test). Except *Tolumonas*, all the other discriminative genera observed in the effluents of SC and ST before disinfection were different. Among them, 10 in SC and 11 in ST were opportunistic pathogens, including *Vibrio* and *Bacteroides* in SC, *Burkholderiaceae, Acinetobacter*, and *Zoogloea* in ST. Some pathogenic genera were also identified as discriminative genera in the disinfected effluents, including *Acinetobacter, Pseudomonas, Shewanella*, and *Flavobacterium* in SC and *Aeromonas* in ST. Their relative abundances significantly increased after disinfection (*p* < 0.05). Indeed, these genera were among the top 15 most abundant genera found in the taxonomic profiles. Although such an increase did not necessarily reflect the changes in the actual amounts of these bacteria, the results showed that these opportunistic pathogens were viable after chlorine disinfection and predominant in the microbiomes. Besides, two pathogenic genera (*Sphingomonas, Paracoccus*) and two non-pathogenic genera (*Denitromonas* and *Sulfurovum*) were found to be prevalent after disinfection in both SC and ST, showing that they were relatively resistant to chlorine disinfection regardless of the types of effluents.

**Figure 4:**
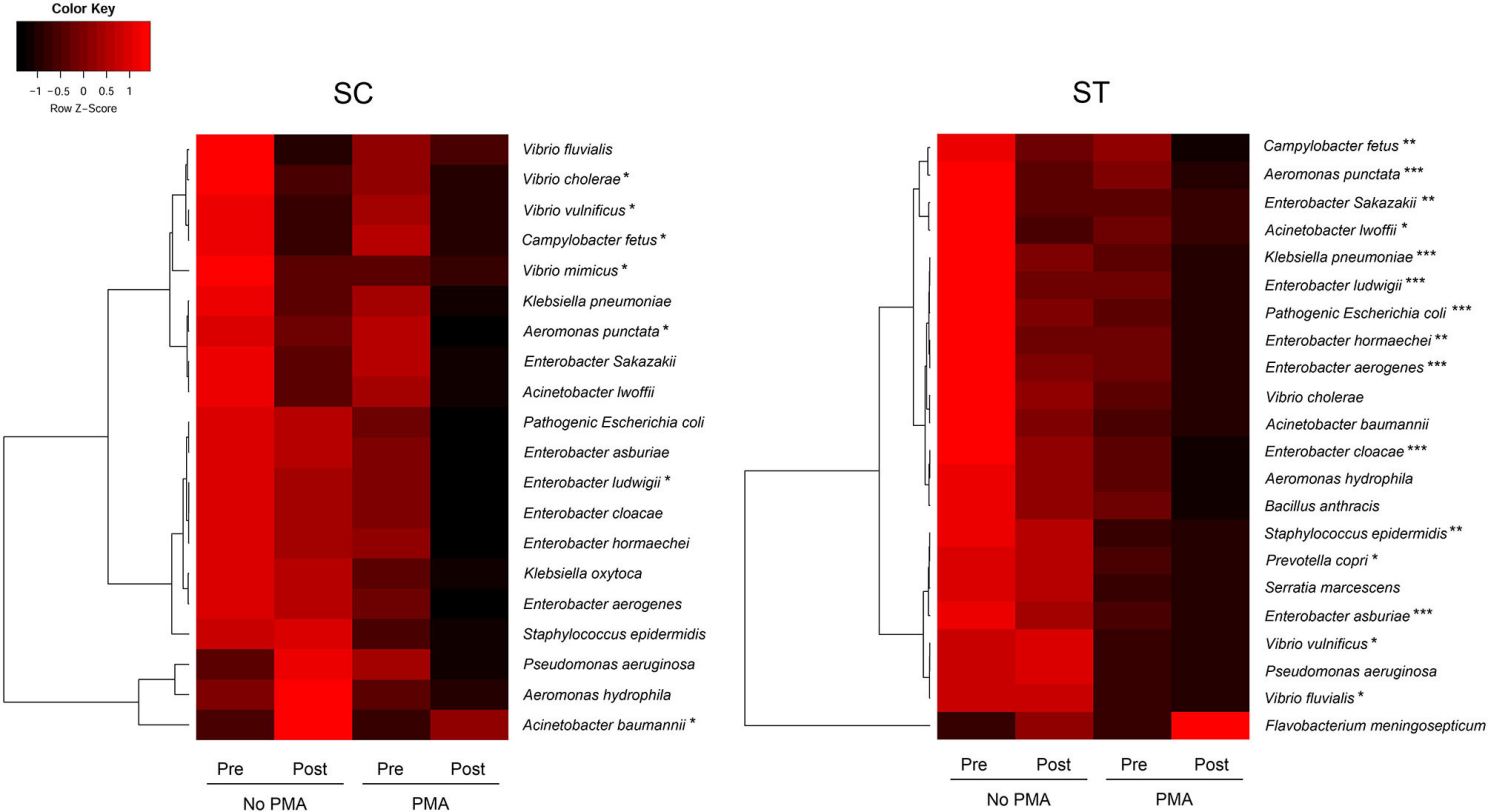
The average concentrations of total and viable pathogenic species before and after chlorine disinfection in each sewage treatment work over the year. Color scale represented the Z-scores in rows. Paired *t-*test for comparing the concentrations of species in all PMA-treated samples before and after chlorine disinfection: *p < 0.05, **p < 0.01, ***p< 0.001.

The pathogen identification analysis pipeline revealed 20 and 22 clinically important pathogenic species with relative abundances of ≥ 0.01 % in the whole bacterial communities of the pre-disinfected samples of SC and ST, respectively (Figure 4). There were 18 species found in both SC and ST, six of them were members of the genus *Enterobacter*, including *E. aerogenes, E. asburiae, E. cloacae, E. hormaechei, E. ludwigii*, and *E. Sakazakii*. Several pathogenic species such as *Aeromonas, Acinetobacter*, and *Vibrio* were affiliated to the top 15 dominant genera aforementioned. The relative abundances of the identified pathogens ranged from 0.01 to 36.21 % among all samples. Except for *Acinetobacter baumannii* in SC and *Flavobacterium meningosepticum* in ST, the estimated concentrations of all other pathogens reduced after chlorine disinfection on average over the year, especially in the viable population. The average reductions of viable pathogens in SC and ST were 0.43 ± 0.24 and 0.53 ± 0.30 log_10_ 16S rRNA gene copies ml^−1^, respectively. There were 15 pathogenic species in ST significantly reduced after disinfection in the viable population, but only 6 species in SC had a significant reduction in the viable population (*p* < 0.05, paired *t*-test). Several species had significant reductions in the viable populations of both SC and ST, including *Aeromonas punctata, Campylobacter fetus, Enterobacter ludwigii*, and *Vibrio vulnificus* (*p* < 0.05, paired t-test). The ranges of concentrations of the pathogenic species in the viable populations of SC were 6.45 to 9.27 log_10_ 16S rRNA copies ml^−1^ before disinfection and 6.24 to 8.72 log_10_ 16S rRNA copies ml^−1^ after disinfection, while that of ST were 3.73 to 6.42 log_10_ 16S rRNA copies ml^−1^ before disinfection and 3.52 to 6.07 log_10_ 16S rRNA copies ml^−1^ after disinfection.

### Correlations Between Total Pathogen Loads and Fecal Indicator Bacteria

**Figure 5:**
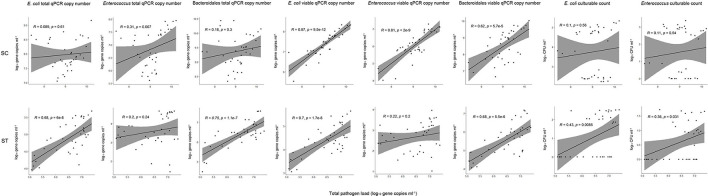
Pearson correlations between total pathogen loads and fecal indicator bacteria with different measurement strategies. The culturable plate counts, total and viable qPCR gene copies of *E. coli, Enterococcus*, and Bacteroidales were compared to the total pathogen loads (all viable pathogens) for all samples collected before and after chlorine disinfection throughout the year in each sewage treatment work.

The sum of all estimated concentrations of the viable pathogenic species in each sample showed different strengths of correlations with the culturable plate counts and total and viable qPCR gene copies of indicator bacteria (Figure 5). For SC, the average concentrations of the pathogen loads of all samples were 9.23 ± 0.57 log_10_ 16S rRNA gene copies ml^−1^ before disinfection and 8.92 ± 0.66 log_10_ 16S rRNA gene copies ml^−1^ after disinfection. For ST, the average concentrations of pathogen loads were 6.81 ± 0.43 log_10_ 16S rRNA gene copies ml^−1^ before disinfection and 6.21 ± 0.75 log_10_ 16S rRNA gene copies ml^−1^ after disinfection.

The culturable counts of *E. coli* and *Enterococcus* had no significant correlations (*p* > 0.05, Pearson correlation) with the pathogen loads in SC samples, and weak correlations (Pearson's *r* < 0.5) with the pathogen loads in ST samples. Even when the number of culturable cells reduced to zero after chlorine disinfection, the gene copies of viable pathogens did not decrease accordingly. In contrast, the concentrations of viable fecal indicators determined by PMA-qPCR showed the best correlations with the pathogen loads. The viable rRNA gene copies of all three indicators (*E. coli, Enterococcus*, and Bacteroidales) had strong correlations with the pathogen loads in SC. The correlation with the viable gene copies of *E. coli* was the strongest (Pearson's *r* = 0.87, *p* < 0.001) in SC samples, followed by *Enterococcus* (Pearson's r = 0.81, p < 0.001) and then Bacteroidales (Pearson's *r* = 0.62, *p* < 0.001). The viable gene copies of *E. coli* and Bacteroidales in ST samples also had significant correlations (p < 0.001), with Pearson's *r* values of 0.7 and 0.68, respectively. However, the viable gene copy numbers of *Enterococcus* in ST did not have a significant correlation with the pathogen loads. It is because the viable gene copies of *Enterococcus* were relatively low in ST samples, only 2.68 log_10_ gene copies ml^−1^ of effluents on average. The measured copies of *Enterococcus* in some of the ST samples were close to the detection limit of qPCR (1 log_10_ gene copies μl^−1^ of the qPCR reaction mixture), which in turn increased the error of the detected copy numbers and weakened the correlations with pathogen loads. For the total qPCR copy numbers (without PMA treatment), all three indicator bacteria had relatively weak (Pearson's *r* < 0.5) or insignificant (*p* > 0.05) correlations in SC, only *E. coli* and Bacteroidales in the ST samples had significant correlations with the pathogen loads (*p* < 0.001).

## Discussion

In this study, chlorine disinfection resulted in a significant drop in the culturable populations of *E. coli* and *Enterococcus* in both sewage treatment works, but the quantitation by PMA-qPCR revealed a considerable amount of viable cells remaining after disinfection. Even when the culturable cells of *E. coli* and *Enterococcus* were undetectable after disinfection, there remained over 3 log_10_ cell ml^−1^ of viable cells in the disinfected effluents (Figure 1A). The differences between the concentrations of viable cell equivalents and culturable counts are in part attributable to the presence of VBNC bacteria. Instead of being completely deactivated by disinfection, VBNC cells may resuscitate after the alleviation of stress factors (e.g., disinfectant) (Oliver et al., 2005; Pienaar et al., 2016; Lin et al., 2017). Pathogens in the VBNC state still carry virulence genes and remain infectious after resuscitation in suitable hosts or environment. In line with previous reports, the results in this study suggested that the current practices of culture-based methods adopted by regulatory agencies are prone to the underestimation of the concentrations of bacterial indicators that remained alive in the disinfected effluents and hence the overestimation of the disinfection efficacy. From the public health perspective, the disinfected effluent may pose a higher bacteriological risk than the results of culture-based methods in effluent monitoring suggest.

Substantial compositional differences were observed between the bacterial communities of the effluents in SC and ST. The operational differences between SC and ST appeared to be the main factor causing the compositional differences, as indicated by the strong correlations between bacterial community structures and physicochemical parameters in the samples (Supplementary Figure 4). Among all the measured abiotic factors, salinity appeared to be the strongest factor differentiating the bacterial community compositions of the two types of effluents. Although the bacterial communities differed markedly in the two contrasting types of effluents, chlorine disinfection significantly reduced the richness, diversity, and evenness of the viable bacterial communities in the effluents of both sewage treatment works (Figure 2). The LEfSe analysis indicated that the viable bacterial populations in the effluents of each sewage treatment work were characterized by specific types of bacteria with most of the representative genera being exclusive to SC or ST before disinfection (Figure 3). However, four representative bacteria genera that remained after disinfection appeared to be common in both SC and ST, including *Sphingomonas, Denitromonas, Paracoccus*, and *Sulfurovum*. This result suggests that chlorine disinfection imposed a selective pressure on the bacterial communities in the effluents irrespective of the influent types and treatment conditions in the two sewage treatment works. More importantly, numerous pathogenic species were found viable after disinfection, many of them belonged to the genera *Enterobacter* and *Vibrio* (Figure 4). Although the concentrations of the viable pathogenic species were reduced after chlorine disinfection, their concentrations remained over 8 log_10_ 16S rRNA copies ml^−1^ in SC and 6 log_10_ 16S rRNA copies ml^−1^ in ST. It indicates that the current practices of disinfection processes could not eliminate these chlorine-resistant pathogens even though the culturable counts of *E. coli* or *Enterococcus* were reduced to below detection level.

Given that numerous types of pathogens remained viable in the effluents after disinfection, we considered the correlations between indicators and the overall pathogen reduction. Our results showed that culture-based detection of *E. coli* and *Enterococcus* had a poor performance as a proxy for the reduction in total pathogen load (Figure 5). Some of the samples with undetectable culturable counts of the indicator bacteria indeed had considerable amounts of viable pathogens. In contrast, the viable concentrations of indicator bacteria measured in PMA-qPCR showed the best correlations with the pathogen loads. All three indicator bacteria tested (*E. coli, Enterococcus*, and Bacteroidales) have moderate to strong correlations with the pathogen loads (Pearson's *r* > 0.6, *p* < 0.001) except for *Enterococcus* in ST (*p* > 0.05). The disparity between the performances of culturable and viable indicator bacteria indicated that the presence of VBNC cells significantly weakens the correlations between the indicator bacteria and pathogens. Overall, the 23S rRNA gene copy numbers of viable *E. coli* showed the best correlations in both sewage treatment works (*p* < 0.001, Pearson's *r* = 0.87 in SC, Pearson's *r* = 0.7 in ST) compared with the other two indicators in this study. The insignificant correlation between viable *Enterococcus* and total pathogen load in ST could be due to the low concentrations of *Enterococcus* in the effluents. The low copy numbers of viable *Enterococcus* in ST may have increased the detection error in qPCR and subsequently impacted the correlation with the pathogen load. In other words, the strength of correlation can be influenced by the scarcity of the bacterial indicator in effluents and the detection limits of the qPCR assays. Interestingly, the viable gene copies of *Enterococcus* had a strong correlation with the pathogen load in SC. This result supported the use of *Enterococcus* as an indicator in saline environments, such as seawater. Due to its great salt tolerance, *Enterococcus* has been widely used as a fecal indicator in beach water (Harwood et al., 2000; Byappanahalli et al., 2012). The results showed that the PMA-qPCR assay of *Enterococcus* had comparable performance to the *E. coli* in a saline effluent. While in the context of freshwater, *E. coli* had a more stable performance than *Enterococcus*.

To minimize public health risks and conserve the quality of receiving waters, the disinfection process should be optimized for pathogen removal, such as combining with an alternative disinfection method or adjusting the dosage and reaction period of chlorination. However, the adjustment of the disinfection process is often a challenging task given the constraints in cost, space, and the production of toxic disinfection by-products (Richardson, 2003). From a public health perspective, it is a critical issue for regulatory authorities to have a reliable indicator to determine the potential threats associated with viable pathogens in effluent discharge. The relationship between the concentrations of indicator bacteria and pathogens is of utmost importance when determining whether a monitoring regime is effective in health risk assessment. This work demonstrated that the choices of fecal indicators and measurement methods have a profound impact on the assessment of disinfection efficacy and the prediction of the health risks associated with pathogens. Over the decades, culture-based measurements of *E. coli* and *Enterococcus* have been routinely used by environmental agencies around the world in the assessment of water sanitation (Price and Wildeboer, 2017), while quantitative PCR assays of host-specific Bacteroidales markers serve as emerging indicators for the tracking of pollution sources (Schriewer et al., 2010; Nshimyimana et al., 2017). Yet, none of these methods can provide an accurate assessment of the health risks upon the discharge of sewage effluents. Amplicon sequencing of the 16S rRNA gene allows us to gain insight into the whole bacterial communities and reveal the diversity and relative abundances of bacterial pathogens in the disinfected effluents. However, as limited by the cost, time for processing, and challenges with potential errors (amplification biases in sequencing), it is unlikely to apply amplicon sequencing as a routine assessment of effluent quality. Nonetheless, it provides a good foundation for investigating the efficacy of different monitoring strategies. In this study, we presented a method using amplicon sequencing and qPCR analysis coupled with PMA treatment to quantify the total amount of viable pathogens in the effluents to evaluate the performances of different types and populations of indicator bacteria. We showed that the concentrations of the viable populations of indicator bacteria not only reflect the actual disinfection efficacy in bacterial removal but also help predict the total pathogen loads in effluents. In fact, the application of viability qPCR methods to quantify the bacterial concentration in sewage requires rigorous assay design. Incomplete signal suppression by PMA, DNA loss during extraction, and amplification inhibition in qPCR are factors that lead to biased estimation of the resulting bacterial gene copy numbers. The efficiency of PMA treatment in intercalating non-viable cell DNA and the DNA recovery during the DNA extraction process can be determined by the use of DNA controls. The copy numbers of gene markers in individual samples obtained from qPCR need to be corrected by using the data of PMA treatment and DNA extraction efficiencies. In addition, the effects of PCR inhibitors in DNA samples can be evaluated by using a serial dilution of the samples. As shown in the study, variance in the DNA extraction and PMA efficiencies was observed across samples, especially between different types of sewage effluents. High salt, nitrogen, and phosphorus content in the sample may adversely affect DNA extraction efficiency. PMA treatment efficiency was particularly lower in SC samples, which contained much more suspended solids. One of the possible reasons may be due to lower light transmittance during the photoactivation of PMA dye.

For the design of the PMA-qPCR assays, another important criterion is the selection of primers and probes for each bacterial target. High coverage and specificity are primary considerations. To enable a fast amplification process and reduce the chance of polymerization error, short amplicons (<200 bp) are preferred. However, shorter amplicons increase the chance of false-positive detection of non-viable cell DNA due to the insufficient binding of PMA molecules to short DNA fragments (Van Holm et al., 2021). In this study, salmon testes DNA (77bp) as the control to quantify the PMA treatment efficiencies had an amplicon length shorter than those of the bacterial gene targets (Table 1). It reduced the chances of overestimating the viable copies in PMA-qPCR assays. Nonetheless, the variance of amplicon length can lead to the different sensitivity of the viability assays, the evaluation of signal suppression using the PMA control does not necessarily indicate the same extent in other PMA-qPCR assays. Therefore, the accuracy in PMA-qPCR quantification of the gene markers of fecal indicators can be affected by the variances of amplicon sizes. This may affect the enumeration of VBNC cells when comparing the difference between viable and culturable concentrations. Since the number of gene copies in the bacterial target varies between species, the conversion of qPCR gene copies to cell equivalent is another source of error in the quantification of VBNC cells.

This study showed the potential of PMA-qPCR as a biological index for monitoring the health risks of effluent discharge. For practical use, since the abundances and survival rates of indicator bacteria vary greatly across different types of effluents, it is important to understand the characteristics of influents and treatment processes, and their subsequent effects on the microbiomes and bacterial indicators, in order to choose the most reliable index in effluent quality monitoring.

## Conclusion

- The quantification of indicator bacteria by PMA-qPCR and cultivation suggested that a large number of VBNC cells were present in disinfected effluents
- Evaluation of disinfection outcome using culture-based quantification of indicator bacteria provides inaccurate information about the reduction of pathogen load and hence the potential biological risks of effluents upon the discharge to receiving waters
- PMA-qPCR quantification of indicator bacteria showed strong correlations with the total pathogen loads in the effluents, with *E. coli* showing the best performance

## Data Availability Statement

The datasets presented in this study can be found in online repositories. The names of the repository/repositories and accession number(s) can be found at: https://www.ncbi.nlm.nih.gov/, PRJNA796191.

## Author Contributions

MT performed the experiments, formal analysis, data visualization, and writing of the original draft. SL provided the resources, supervision, administration and funding acquisition of the project, contributed to the validation, and review of the manuscript. All authors contributed to the conceptualization, methodology and data curation of the study.

## Funding

This study was financially supported by the General Research Fund Scheme of the Research Grants Council of Hong Kong (project no. 16301018) and the Theme-based Research Scheme (T21-602/16-R) of the University Grants Committee of Hong Kong.

## Conflict of Interest

The authors declare that the research was conducted in the absence of any commercial or financial relationships that could be construed as a potential conflict of interest.

## Publisher's Note

All claims expressed in this article are solely those of the authors and do not necessarily represent those of their affiliated organizations, or those of the publisher, the editors and the reviewers. Any product that may be evaluated in this article, or claim that may be made by its manufacturer, is not guaranteed or endorsed by the publisher.
