# Supplementary material for: Strategy to Evaluate Changes in Bacterial Community Profiles and Bacterial Pathogen Load Reduction After Sewage Disinfection
Source: Front Microbiol. 2022 Jul 11;13:919207. doi: 10.3389/fmicb.2022.919207 (PMC9309643; doi:10.3389/fmicb.2022.919207)
Supplement: Supplementary file 1 [file Data_Sheet_1.DOCX]

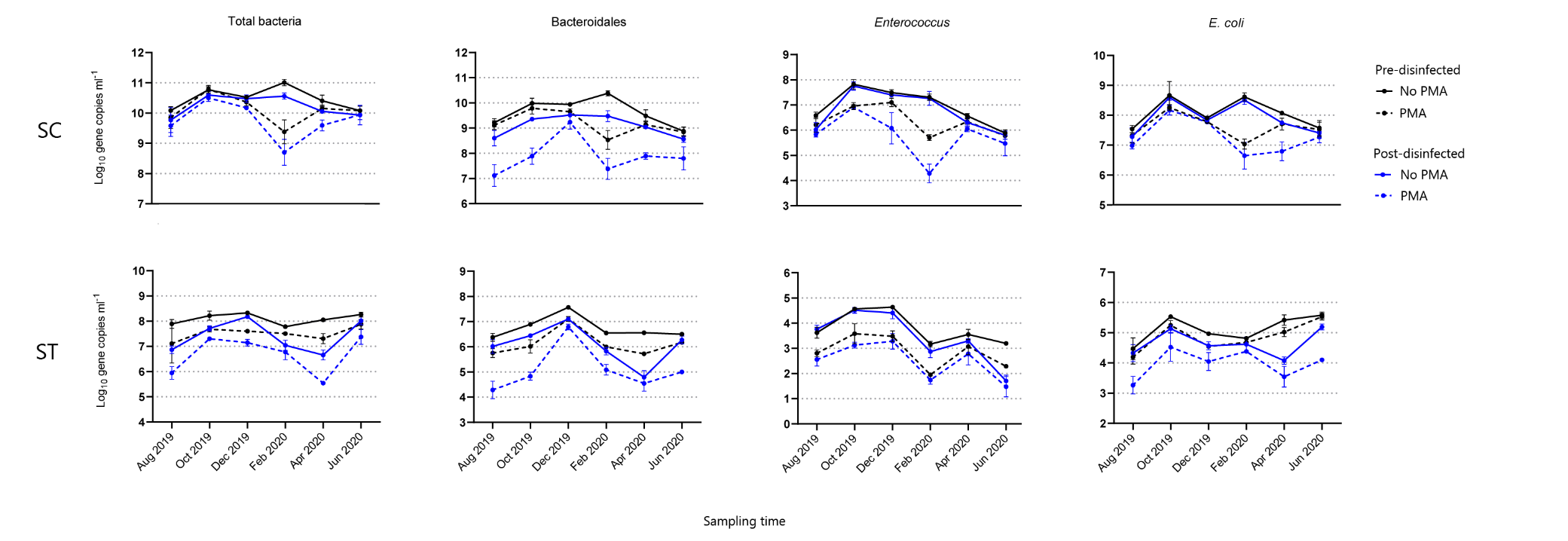


**Figure S1.** Total and viable concentrations of total bacteria, Bacteroidales, *Enterococcus* and *E. coli* before and after chlorination in each STW throughout a year. The concentrations of total (no PMA) and viable (PMA) cells in each group of bacteria were determined by qPCR assays and corrected with DNA extraction and PMA treatment efficiencies. Data are shown as mean ± 1 S.D. log_10_ copies per ml of effluent derived from the three biological replicates.


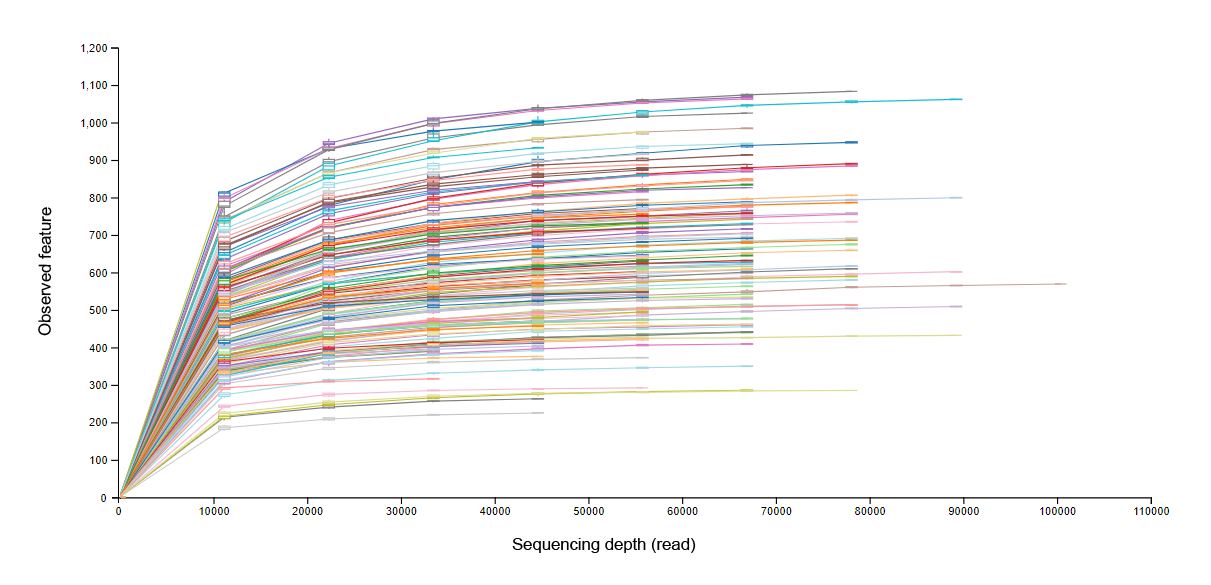


**Figure S2.** Alpha rarefaction curve of observed features (sequence variants) against sequencing depths. The numbers of observed features at each sampling depth were computed for all samples.


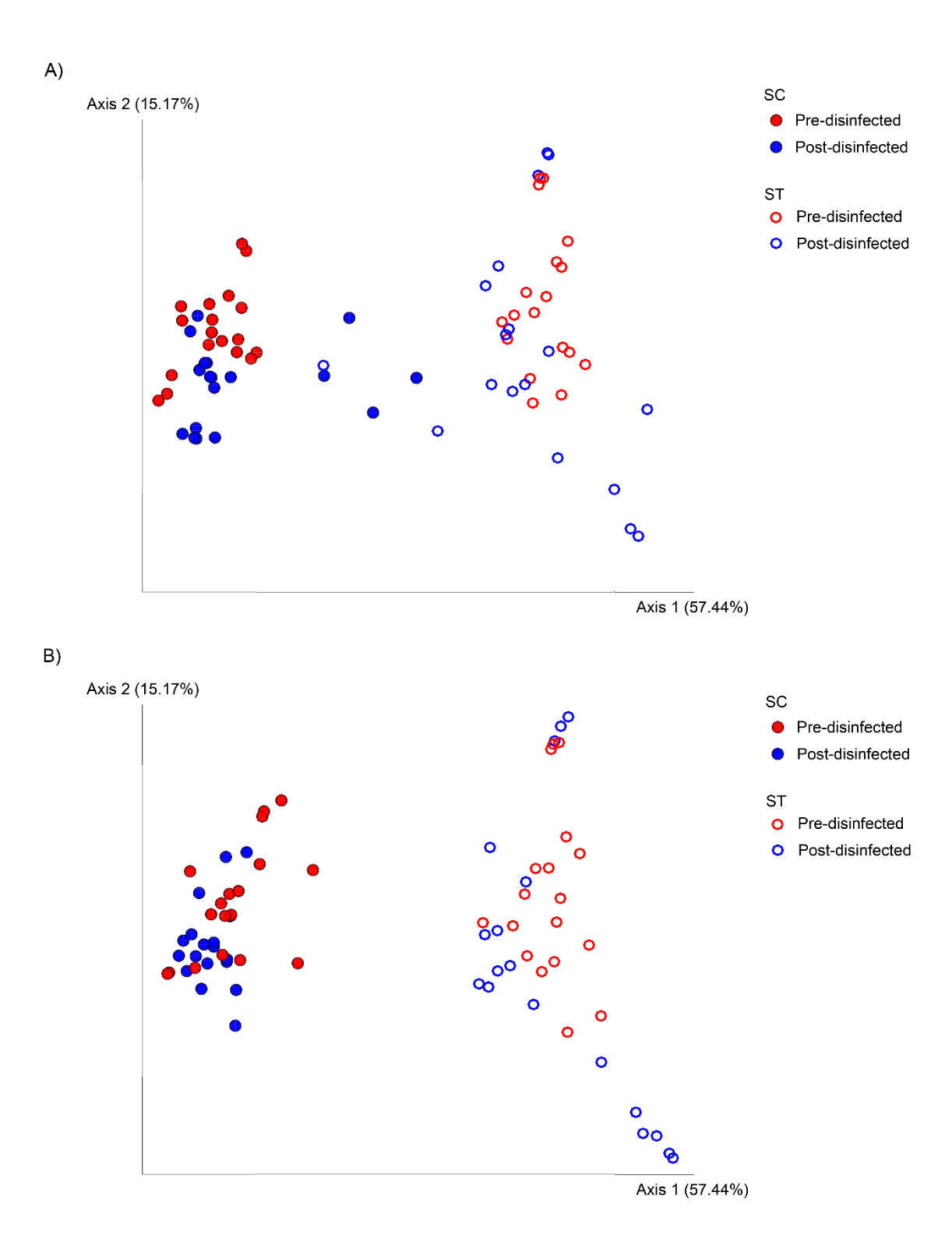


**Figure S3.** Principal Coordinates Analysis (PCoA) plot illustrating the community dissimilarity along axis 1 and 2 based on weighted UniFrac distance. **(A)** Total bacterial community and **(B)** Viable bacterial community. All samples were first rarefied to the minimum depth of filtered reads, the sequence variants observed in each sample were then used for the calculation. The percentage of variation explained by each principal coordinate was indicated on the axes.


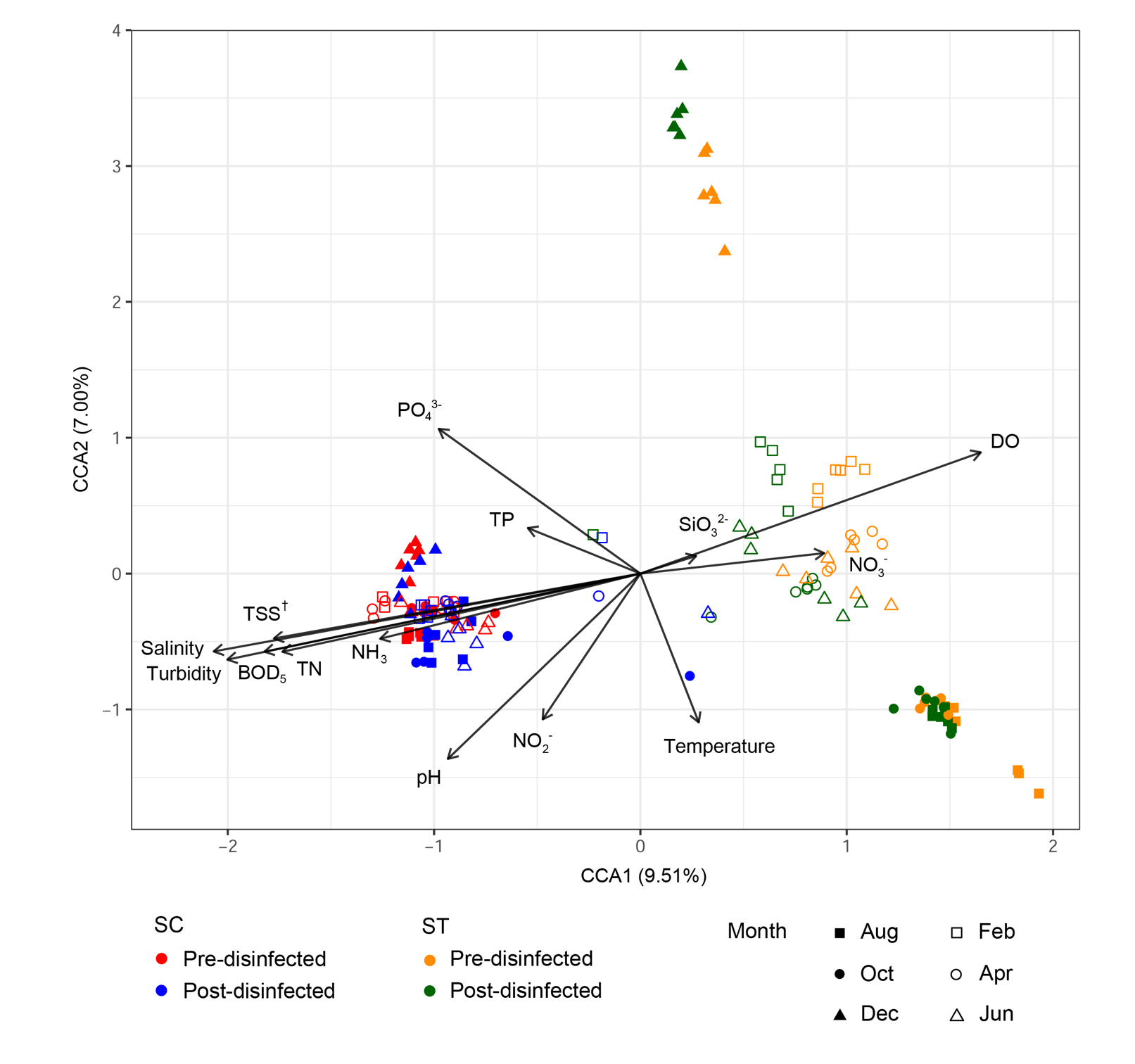


**Figure S4.** Constrained Correspondence Analysis (CCA) plot showing the correlations between bacterial community structures and physicochemical parameters. It was constructed based on the Chi-squared distance matrices of sequence variants compositions explained by the physicochemical parameters. The direction of arrow pointed towards the increase in amount of variable, and the length of arrow was relative to the strength of correlation with the variable. All variables were statistically significant (p < 0.05) except TSS (marked with †). The percentages of variation explained by each constrained axis were indicated.


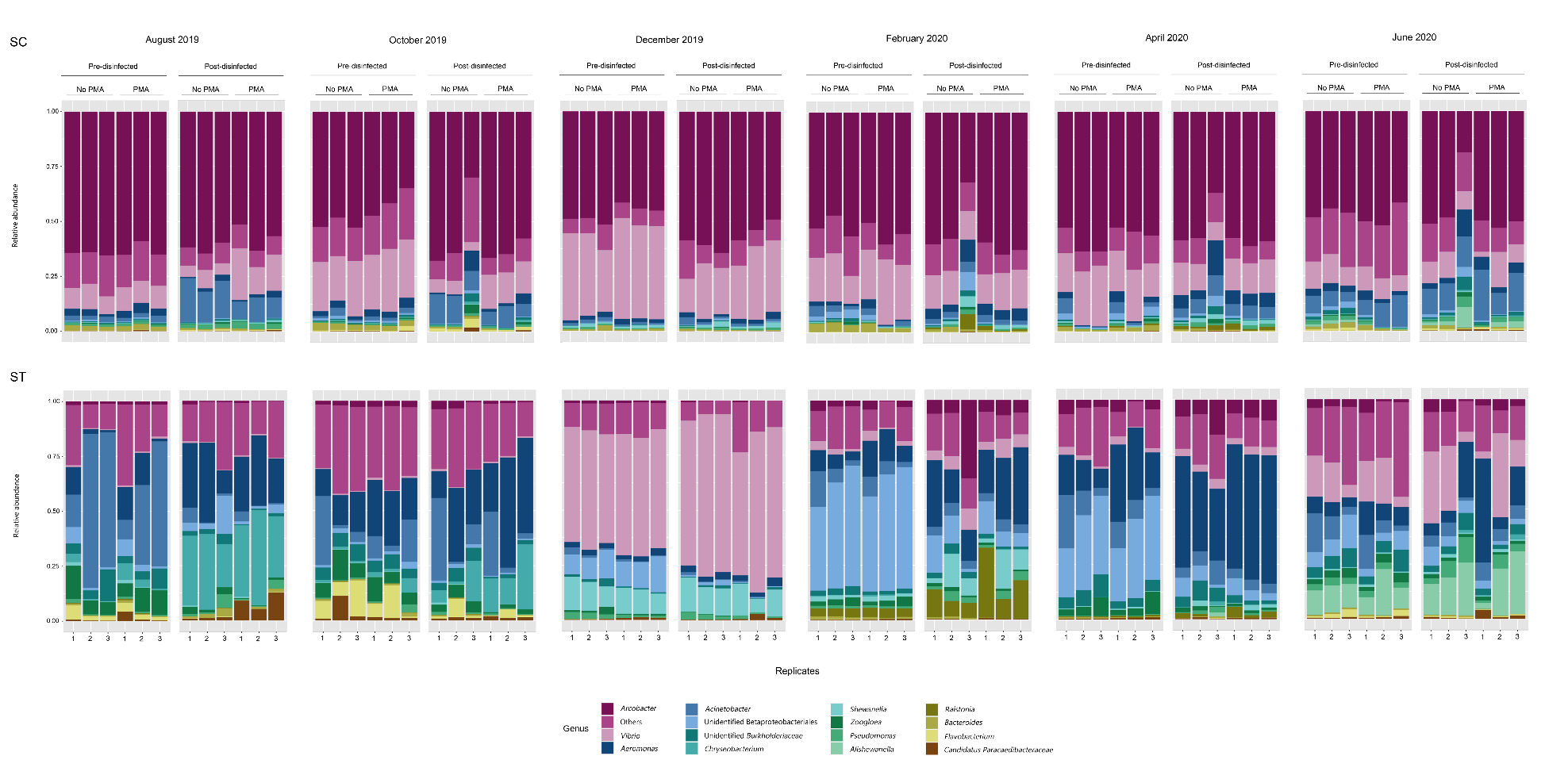


**Figure S5**. Taxa plots of the top 15 abundant genera in two STWs throughout the year. The relative abundance of each taxa refers to its mapped count divided by the total bacterial counts. The number below each bar indicated the three biological replicates before and after disinfection.

**Table S1.** Operational and physicochemical parameters of the effluents in SC and ST.

*The data of total residual chlorine during sample collection were provided by the sewage treatment facilities

^†^ Measurements were conducted on site or in the laboratory after sample collection. The details of methods were described in ‘Physicochemical Parameters’ section in Materials and Methods. Values are expressed as mean ± 1 S.D.

| Operational and physicochemical parameters | SC | ST |
| --- | --- | --- |
| Treatment capacity (m^3^/day) | 1.90E+06 | 8.80E+03 |
| Influent | Saline | Freshwater |
| Screenings and degritting | ✓ | ✓ |
| Chemically enhanced primary treatment | ✓ | ☓ |
| Secondary treatment | ☓ | ✓ |
| Total residual chlorine (mg/L) * | < 0.1 | 0.2 - 0.4 |
| Temperature (^o^C) ^†^ | 26.79 ± 2.97 | 26.65 ± 2.51 |
| Salinity (ppt) ^†^ | 13.42 ± 1.57 | 0.14 ± 0.03 |
| Dissolved oxygen (mg/L) ^†^ | 1.91 ± 0.75 | 4.26 ± 0.69 |
| pH ^†^ | 7.45 ± 0.52 | 6.72 ± 0.49 |
| Turbidity (NTU) ^†^ | 68.74 ± 30.79 | 1.86 ± 0.55 |
| Biological oxygen demand (mg/L) ^†^ | 65.06 ± 20.89 | 7.95 ± 4.12 |
| Total suspended solids (mg/L) ^†^ | 65.73 ± 25.81 | 5.51 ± 3.25 |
| Total nitrogen (mg/L) ^†^ | 23.33 ± 8.21 | 4.22 ± 1.41 |
| Ammonia (mg/L) ^†^ | 10.44 ± 9.58 | 0.25 ± 0.12 |
| Nitrite (mg/L) ^†^ | 0.03 ± 0.01 | 0.01 ± 0.02 |
| Nitrate (mg/L) ^†^ | 0.07 ± 0.04 | 0.27 ± 0.27 |
| Total phosphorous (mg/L) ^†^ | 4.07 ± 3.64 | 2.60 ± 2.43 |
| Phosphate (mg/L) ^†^ | 1.10 ± 0.38 | 0.83 ± 0.42 |
| Silicate (mg/L) ^†^ | 1.51 ± 0.38 | 1.63 ± 0.69 |

**Table S2.** Dilution used for qPCR assays**,** DNA extraction and PMA treatment efficiencies of **(A)** SC samples and **(B)** ST samples.

**(A)**

| Site | Month | Chlorination | PMA treatment | Replicates | Dilution used | Extraction efficiency | PMA efficiency |
| --- | --- | --- | --- | --- | --- | --- | --- |
| SC | Aug | Pre | Yes | 1 | 0 | 4.46% | 78.95% |
|  |  |  |  | 2 | 0 | 1.78% | 79.23% |
|  |  |  |  | 3 | 0 | 2.25% | 63.59% |
|  |  |  | No | 1 | 0 | 2.20% | - |
|  |  |  |  | 2 | 0 | 2.87% | - |
|  |  |  |  | 3 | 0 | 1.88% | - |
|  |  | Post | Yes | 1 | 0 | 7.89% | 86.41% |
|  |  |  |  | 2 | 0 | 4.27% | 62.16% |
|  |  |  |  | 3 | 0 | 2.55% | 83.22% |
|  |  |  | No | 1 | 0 | 3.97% | - |
|  |  |  |  | 2 | 0 | 6.82% | - |
|  |  |  |  | 3 | 0 | 1.71% | - |
|  | Oct | Pre | Yes | 1 | 10x | 0.15% | 90.88% |
|  |  |  |  | 2 | 10x | 0.32% | 83.76% |
|  |  |  |  | 3 | 0 | 0.53% | 93.55% |
|  |  |  | No | 1 | 0 | 0.22% | - |
|  |  |  |  | 2 | 10x | 0.23% | - |
|  |  |  |  | 3 | 10x | 0.52% | - |
|  |  | Post | Yes | 1 | 10x | 0.30% | 92.78% |
|  |  |  |  | 2 | 10x | 0.21% | 93.63% |
|  |  |  |  | 3 | 10x | 0.47% | 93.15% |
|  |  |  | No | 1 | 10x | 0.13% | - |
|  |  |  |  | 2 | 0 | 0.15% | - |
|  |  |  |  | 3 | 0 | 0.29% | - |
|  | Dec | Pre | Yes | 1 | 0 | 1.22% | 87.04% |
|  |  |  |  | 2 | 0 | 1.25% | 70.68% |
|  |  |  |  | 3 | 0 | 1.10% | 87.21% |
|  |  |  | No | 1 | 0 | 1.23% | - |
|  |  |  |  | 2 | 0 | 0.91% | - |
|  |  |  |  | 3 | 0 | 0.98% | - |
|  |  | Post | Yes | 1 | 0 | 1.26% | 84.97% |
|  |  |  |  | 2 | 10x | 1.25% | 77.28% |
|  |  |  |  | 3 | 0 | 1.18% | 71.17% |
|  |  |  | No | 1 | 0 | 1.16% | - |
|  |  |  |  | 2 | 0 | 1.20% | - |
|  |  |  |  | 3 | 10x | 0.82% | - |
|  | Feb | Pre | Yes | 1 | 0 | 1.53% | 91.59% |
|  |  |  |  | 2 | 0 | 1.64% | 89.48% |
|  |  |  |  | 3 | 0 | 1.95% | 93.59% |
|  |  |  | No | 1 | 0 | 0.20% | - |
|  |  |  |  | 2 | 10x | 0.24% | - |
|  |  |  |  | 3 | 0 | 0.18% | - |
|  |  | Post | Yes | 1 | 10x | 0.55% | 90.28% |
|  |  |  |  | 2 | 0 | 1.64% | 90.59% |
|  |  |  |  | 3 | 0 | 1.68% | 90.67% |
|  |  |  | No | 1 | 10x | 0.06% | - |
|  |  |  |  | 2 | 0 | 0.20% | - |
|  |  |  |  | 3 | 10x | 0.19% | - |
|  | Apr | Pre | Yes | 1 | 10x | 1.02% | 82.33% |
|  |  |  |  | 2 | 10x | 1.03% | 82.36% |
|  |  |  |  | 3 | 0 | 1.39% | 73.51% |
|  |  |  | No | 1 | 10x | 0.93% | - |
|  |  |  |  | 2 | 10x | 0.75% | - |
|  |  |  |  | 3 | 0 | 0.75% | - |
|  |  | Post | Yes | 1 | 10x | 0.98% | 83.79% |
|  |  |  |  | 2 | 0 | 1.23% | 85.89% |
|  |  |  |  | 3 | 0 | 1.13% | 82.84% |
|  |  |  | No | 1 | 0 | 1.16% | - |
|  |  |  |  | 2 | 0 | 1.19% | - |
|  |  |  |  | 3 | 10x | 0.93% | - |
|  | Jun | Pre | Yes | 1 | 0 | 1.99% | 61.71% |
|  |  |  |  | 2 | 0 | 1.76% | 74.80% |
|  |  |  |  | 3 | 0 | 1.73% | 60.87% |
|  |  |  | No | 1 | 0 | 1.77% | - |
|  |  |  |  | 2 | 0 | 1.71% | - |
|  |  |  |  | 3 | 0 | 1.61% | - |
|  |  | Post | Yes | 1 | 0 | 1.99% | 68.17% |
|  |  |  |  | 2 | 0 | 1.68% | 54.36% |
|  |  |  |  | 3 | 10x | 1.62% | 59.39% |
|  |  |  | No | 1 | 10x | 1.11% | - |
|  |  |  |  | 2 | 10x | 1.22% | - |
|  |  |  |  | 3 | 10x | 1.28% | - |

**(B)**

| Site | Month | Chlorination | PMA treatment | Replicates | Dilution used | Extraction efficiency | PMA efficiency |
| --- | --- | --- | --- | --- | --- | --- | --- |
| ST | Aug | Pre | Yes | 1 | 10x | 8.93% | 99.98% |
|  |  |  |  | 2 | 10x | 7.74% | 99.98% |
|  |  |  |  | 3 | 0 | 9.54% | 99.98% |
|  |  |  | No | 1 | 0 | 4.01% | - |
|  |  |  |  | 2 | 10x | 7.53% | - |
|  |  |  |  | 3 | 0 | 8.78% | - |
|  |  | Post | Yes | 1 | 10x | 7.28% | 99.97% |
|  |  |  |  | 2 | 0 | 6.30% | 99.96% |
|  |  |  |  | 3 | 0 | 3.09% | 99.94% |
|  |  |  | No | 1 | 10x | 3.55% | - |
|  |  |  |  | 2 | 0 | 7.01% | - |
|  |  |  |  | 3 | 10x | 3.36% | - |
|  | Oct | Pre | Yes | 1 | 0 | 1.44% | 97.95% |
|  |  |  |  | 2 | 0 | 1.34% | 99.46% |
|  |  |  |  | 3 | 0 | 1.30% | 99.54% |
|  |  |  | No | 1 | 0 | 1.45% | - |
|  |  |  |  | 2 | 0 | 1.58% | - |
|  |  |  |  | 3 | 0 | 1.33% | - |
|  |  | Post | Yes | 1 | 0 | 1.22% | 99.36% |
|  |  |  |  | 2 | 0 | 1.47% | 98.45% |
|  |  |  |  | 3 | 0 | 1.26% | 99.47% |
|  |  |  | No | 1 | 0 | 1.33% | - |
|  |  |  |  | 2 | 0 | 1.36% | - |
|  |  |  |  | 3 | 0 | 1.12% | - |
|  | Dec | Pre | Yes | 1 | 10x | 1.42% | 99.86% |
|  |  |  |  | 2 | 10x | 1.06% | 99.83% |
|  |  |  |  | 3 | 0 | 1.33% | 99.88% |
|  |  |  | No | 1 | 0 | 1.36% | - |
|  |  |  |  | 2 | 10x | 1.08% | - |
|  |  |  |  | 3 | 0 | 1.12% | - |
|  |  | Post | Yes | 1 | 10x | 1.33% | 99.80% |
|  |  |  |  | 2 | 10x | 0.76% | 99.87% |
|  |  |  |  | 3 | 0 | 1.07% | 99.83% |
|  |  |  | No | 1 | 0 | 1.05% | - |
|  |  |  |  | 2 | 0 | 1.04% | - |
|  |  |  |  | 3 | 0 | 1.14% | - |
|  | Feb | Pre | Yes | 1 | 0 | 3.23% | 99.92% |
|  |  |  |  | 2 | 0 | 3.26% | 99.90% |
|  |  |  |  | 3 | 0 | 3.19% | 99.91% |
|  |  |  | No | 1 | 0 | 3.46% | - |
|  |  |  |  | 2 | 0 | 3.10% | - |
|  |  |  |  | 3 | 0 | 2.92% | - |
|  |  | Post | Yes | 1 | 0 | 3.10% | 99.87% |
|  |  |  |  | 2 | 0 | 1.05% | 99.86% |
|  |  |  |  | 3 | 0 | 1.74% | 99.91% |
|  |  |  | No | 1 | 0 | 2.07% | - |
|  |  |  |  | 2 | 0 | 1.79% | - |
|  |  |  |  | 3 | 0 | 1.86% | - |
|  | Apr | Pre | Yes | 1 | 0 | 1.78% | 99.88% |
|  |  |  |  | 2 | 0 | 2.13% | 99.84% |
|  |  |  |  | 3 | 10x | 1.77% | 99.90% |
|  |  |  | No | 1 | 0 | 1.73% | - |
|  |  |  |  | 2 | 10x | 1.53% | - |
|  |  |  |  | 3 | 0 | 1.98% | - |
|  |  | Post | Yes | 1 | 10x | 0.41% | 99.64% |
|  |  |  |  | 2 | 10x | 1.34% | 99.76% |
|  |  |  |  | 3 | 0 | 1.75% | 99.85% |
|  |  |  | No | 1 | 0 | 0.80% | - |
|  |  |  |  | 2 | 0 | 1.34% | - |
|  |  |  |  | 3 | 0 | 1.50% | - |
|  | Jun | Pre | Yes | 1 | 0 | 2.60% | 99.89% |
|  |  |  |  | 2 | 0 | 2.45% | 99.89% |
|  |  |  |  | 3 | 0 | 2.19% | 99.91% |
|  |  |  | No | 1 | 0 | 2.67% | - |
|  |  |  |  | 2 | 0 | 2.54% | - |
|  |  |  |  | 3 | 0 | 2.49% | - |
|  |  | Post | Yes | 1 | 0 | 2.11% | 99.90% |
|  |  |  |  | 2 | 0 | 2.51% | 99.88% |
|  |  |  |  | 3 | 0 | 2.10% | 99.88% |
|  |  |  | No | 1 | 0 | 2.52% | - |
|  |  |  |  | 2 | 0 | 2.51% | - |
|  |  |  |  | 3 | 0 | 2.11% | - |

**Table S3.** Numbers of raw and filtered reads, Q30 values and GC contents of **(A)** SC samples and **(B)** ST samples.

**(A)**

| Site | Month | Chlorination | PMA treatment | Replicates | Raw reads | Filtered reads | Q30 % | GC % |
| --- | --- | --- | --- | --- | --- | --- | --- | --- |
| SC | Aug | Pre | Yes | 1 | 93251 | 55344 | 94 | 49.18 |
|  |  |  |  | 2 | 95449 | 68392 | 94.11 | 49.53 |
|  |  |  |  | 3 | 95916 | 49768 | 94.14 | 49.16 |
|  |  |  | No | 1 | 92809 | 68086 | 93.75 | 49.3 |
|  |  |  |  | 2 | 90818 | 63996 | 93.87 | 49.39 |
|  |  |  |  | 3 | 100096 | 54334 | 94.31 | 49.06 |
|  |  | Post | Yes | 1 | 100875 | 60866 | 93.85 | 50.03 |
|  |  |  |  | 2 | 105111 | 65892 | 94.05 | 49.4 |
|  |  |  |  | 3 | 101330 | 68451 | 93.56 | 49.83 |
|  |  |  | No | 1 | 106580 | 75129 | 94.04 | 49.32 |
|  |  |  |  | 2 | 104275 | 71014 | 94.02 | 49.28 |
|  |  |  |  | 3 | 109369 | 81446 | 92.9 | 49.57 |
|  | Oct | Pre | Yes | 1 | 102168 | 63708 | 93.58 | 50.06 |
|  |  |  |  | 2 | 105180 | 72269 | 93.72 | 50.2 |
|  |  |  |  | 3 | 102553 | 61825 | 93.75 | 50.71 |
|  |  |  | No | 1 | 109748 | 79101 | 93.94 | 49.88 |
|  |  |  |  | 2 | 96804 | 57312 | 93.89 | 50 |
|  |  |  |  | 3 | 106256 | 69511 | 94.13 | 49.79 |
|  |  | Post | Yes | 1 | 93970 | 56278 | 93.79 | 49.48 |
|  |  |  |  | 2 | 97877 | 69187 | 93.97 | 49.63 |
|  |  |  |  | 3 | 105790 | 52439 | 93.67 | 49.78 |
|  |  |  | No | 1 | 106504 | 63312 | 93.96 | 49.33 |
|  |  |  |  | 2 | 90725 | 56682 | 94.26 | 49.56 |
|  |  |  |  | 3 | 109065 | 52219 | 93.47 | 51.27 |
|  | Dec | Pre | Yes | 1 | 94928 | 41723 | 91.39 | 50.88 |
|  |  |  |  | 2 | 96685 | 38649 | 92.6 | 50.69 |
|  |  |  |  | 3 | 100009 | 34683 | 92.42 | 50.63 |
|  |  |  | No | 1 | 97606 | 47945 | 92.75 | 50.38 |
|  |  |  |  | 2 | 96372 | 47498 | 92.63 | 50.38 |
|  |  |  |  | 3 | 94417 | 59128 | 91.91 | 50.17 |
|  |  | Post | Yes | 1 | 105034 | 68082 | 92.08 | 49.97 |
|  |  |  |  | 2 | 99905 | 44869 | 92.62 | 50.13 |
|  |  |  |  | 3 | 104268 | 70248 | 92.56 | 50.53 |
|  |  |  | No | 1 | 100654 | 59616 | 92.3 | 49.89 |
|  |  |  |  | 2 | 98303 | 45315 | 92.9 | 49.78 |
|  |  |  |  | 3 | 91280 | 38142 | 92.96 | 49.47 |
|  | Feb | Pre | Yes | 1 | 157957 | 76142 | 86.96 | 50.35 |
|  |  |  |  | 2 | 151480 | 91795 | 91.03 | 49.85 |
|  |  |  |  | 3 | 165493 | 100383 | 91.05 | 50.49 |
|  |  |  | No | 1 | 133857 | 61087 | 86.71 | 50.1 |
|  |  |  |  | 2 | 144043 | 69195 | 86.86 | 50.38 |
|  |  |  |  | 3 | 130797 | 56580 | 87 | 50.12 |
|  |  | Post | Yes | 1 | 158792 | 73838 | 87.06 | 50.12 |
|  |  |  |  | 2 | 145242 | 62697 | 86.91 | 49.97 |
|  |  |  |  | 3 | 168505 | 76992 | 87.12 | 50.02 |
|  |  |  | No | 1 | 161941 | 73546 | 86.63 | 50.06 |
|  |  |  |  | 2 | 163549 | 77683 | 86.74 | 50.16 |
|  |  |  |  | 3 | 143270 | 62356 | 85.91 | 51.77 |
|  | Apr | Pre | Yes | 1 | 139317 | 65072 | 86.82 | 50.44 |
|  |  |  |  | 2 | 153359 | 97969 | 91.33 | 50.4 |
|  |  |  |  | 3 | 138730 | 74718 | 86.37 | 50.12 |
|  |  |  | No | 1 | 145473 | 66729 | 86.6 | 50.27 |
|  |  |  |  | 2 | 147851 | 85768 | 91.01 | 50.06 |
|  |  |  |  | 3 | 134150 | 80754 | 91.38 | 49.92 |
|  |  | Post | Yes | 1 | 154992 | 75452 | 86.84 | 50.21 |
|  |  |  |  | 2 | 137627 | 64200 | 86.02 | 50.14 |
|  |  |  |  | 3 | 164359 | 78618 | 86.91 | 50.19 |
|  |  |  | No | 1 | 132994 | 62045 | 86.26 | 50.23 |
|  |  |  |  | 2 | 123523 | 60217 | 85.68 | 50.23 |
|  |  |  |  | 3 | 165233 | 77915 | 86.74 | 51.54 |
|  | Jun | Pre | Yes | 1 | 166473 | 76676 | 86.31 | 50.37 |
|  |  |  |  | 2 | 134217 | 90185 | 90.74 | 50.5 |
|  |  |  |  | 3 | 113717 | 73802 | 91.89 | 51.26 |
|  |  |  | No | 1 | 150844 | 68226 | 86.36 | 50.42 |
|  |  |  |  | 2 | 93339 | 44439 | 86.3 | 50.55 |
|  |  |  |  | 3 | 138911 | 62469 | 85.86 | 50.45 |
|  |  | Post | Yes | 1 | 116153 | 58672 | 86.41 | 50.3 |
|  |  |  |  | 2 | 130398 | 62609 | 85.95 | 50.38 |
|  |  |  |  | 3 | 121683 | 59283 | 86.67 | 50.44 |
|  |  |  | No | 1 | 154069 | 72653 | 86.18 | 50.33 |
|  |  |  |  | 2 | 101760 | 50664 | 86.42 | 50.48 |
|  |  |  |  | 3 | 158651 | 78754 | 86.39 | 52.55 |

| Site | Month | Chlorination | PMA treatment | Replicates | Raw reads | Filtered reads | Q30 % | GC % |
| --- | --- | --- | --- | --- | --- | --- | --- | --- |
| ST | Aug | Pre | Yes | 1 | 93576 | 69768 | 93.13 | 52.28 |
|  |  |  |  | 2 | 100490 | 66321 | 93.17 | 52.5 |
|  |  |  |  | 3 | 106380 | 63970 | 93.27 | 52.21 |
|  |  |  | No | 1 | 102647 | 66567 | 92.98 | 52.65 |
|  |  |  |  | 2 | 92908 | 45114 | 93.11 | 51.66 |
|  |  |  |  | 3 | 99471 | 69180 | 93.26 | 52.37 |
|  |  | Post | Yes | 1 | 90006 | 70271 | 93.17 | 51.91 |
|  |  |  |  | 2 | 104114 | 80251 | 92.98 | 51.47 |
|  |  |  |  | 3 | 108431 | 85155 | 93.19 | 51.76 |
|  |  |  | No | 1 | 102682 | 68356 | 92.45 | 52.01 |
|  |  |  |  | 2 | 100020 | 75738 | 93.11 | 52.05 |
|  |  |  |  | 3 | 100870 | 72686 | 93.09 | 52.1 |
|  | Oct | Pre | Yes | 1 | 104306 | 61541 | 93.44 | 52.85 |
|  |  |  |  | 2 | 93678 | 66584 | 93.3 | 52.26 |
|  |  |  |  | 3 | 108630 | 74879 | 93.36 | 52.41 |
|  |  |  | No | 1 | 106553 | 78136 | 93.35 | 52.12 |
|  |  |  |  | 2 | 106644 | 61544 | 93.69 | 52.89 |
|  |  |  |  | 3 | 95041 | 47494 | 93.36 | 52.23 |
|  |  | Post | Yes | 1 | 100890 | 74053 | 93.29 | 51.65 |
|  |  |  |  | 2 | 93115 | 67300 | 93.39 | 52.72 |
|  |  |  |  | 3 | 100233 | 58460 | 93.2 | 52.11 |
|  |  |  | No | 1 | 101932 | 52894 | 93.47 | 52.13 |
|  |  |  |  | 2 | 90117 | 66969 | 93.33 | 52.97 |
|  |  |  |  | 3 | 105819 | 77857 | 93.45 | 52.57 |
|  | Dec | Pre | Yes | 1 | 102647 | 49714 | 92.43 | 52.51 |
|  |  |  |  | 2 | 101571 | 52430 | 92.35 | 52.49 |
|  |  |  |  | 3 | 91411 | 62601 | 91.79 | 52.57 |
|  |  |  | No | 1 | 97343 | 52303 | 92.07 | 52.82 |
|  |  |  |  | 2 | 106023 | 62499 | 92.34 | 52.82 |
|  |  |  |  | 3 | 91199 | 49638 | 92.12 | 52.85 |
|  |  | Post | Yes | 1 | 92632 | 63341 | 92.21 | 52.36 |
|  |  |  |  | 2 | 102954 | 50499 | 92.11 | 52.57 |
|  |  |  |  | 3 | 107161 | 78613 | 92.67 | 52.88 |
|  |  |  | No | 1 | 99882 | 69706 | 92.55 | 53 |
|  |  |  |  | 2 | 97686 | 47336 | 92.49 | 53.04 |
|  |  |  |  | 3 | 101549 | 69990 | 92.52 | 53.06 |
|  | Feb | Pre | Yes | 1 | 169993 | 80617 | 84.75 | 52.61 |
|  |  |  |  | 2 | 130713 | 66721 | 86.06 | 52.71 |
|  |  |  |  | 3 | 165920 | 86113 | 85.83 | 52.45 |
|  |  |  | No | 1 | 145978 | 68788 | 85.76 | 52.32 |
|  |  |  |  | 2 | 164791 | 83512 | 85.86 | 52.41 |
|  |  |  |  | 3 | 149805 | 76248 | 85.93 | 52.47 |
|  |  | Post | Yes | 1 | 157609 | 80484 | 85.96 | 53.22 |
|  |  |  |  | 2 | 152506 | 67391 | 85.97 | 53.48 |
|  |  |  |  | 3 | 143204 | 69630 | 85.21 | 53.48 |
|  |  |  | No | 1 | 130887 | 63293 | 86.18 | 53.2 |
|  |  |  |  | 2 | 138477 | 67282 | 85.95 | 53.29 |
|  |  |  |  | 3 | 148570 | 70527 | 86.56 | 51.64 |
|  | Apr | Pre | Yes | 1 | 144970 | 72321 | 86.24 | 53.12 |
|  |  |  |  | 2 | 135250 | 70898 | 87.06 | 53.28 |
|  |  |  |  | 3 | 133205 | 70646 | 86.94 | 53.04 |
|  |  |  | No | 1 | 163327 | 80280 | 86.82 | 52.59 |
|  |  |  |  | 2 | 144808 | 73825 | 86.23 | 52.81 |
|  |  |  |  | 3 | 164594 | 84612 | 86.97 | 53.07 |
|  |  | Post | Yes | 1 | 130046 | 72419 | 86.08 | 53.5 |
|  |  |  |  | 2 | 148111 | 81640 | 87 | 53.39 |
|  |  |  |  | 3 | 156585 | 87692 | 86.97 | 53.38 |
|  |  |  | No | 1 | 132209 | 70593 | 87.04 | 53.43 |
|  |  |  |  | 2 | 169824 | 92888 | 86.98 | 53.36 |
|  |  |  |  | 3 | 136670 | 68372 | 85.95 | 52.46 |
|  | Jun | Pre | Yes | 1 | 165743 | 77910 | 86.6 | 53.09 |
|  |  |  |  | 2 | 148894 | 73504 | 86.85 | 53.66 |
|  |  |  |  | 3 | 115480 | 60159 | 86.77 | 53.37 |
|  |  |  | No | 1 | 135602 | 64182 | 86.48 | 53 |
|  |  |  |  | 2 | 130288 | 67191 | 86.7 | 53.12 |
|  |  |  |  | 3 | 144803 | 69453 | 86.42 | 53.08 |
|  |  | Post | Yes | 1 | 160417 | 90838 | 86.76 | 53.59 |
|  |  |  |  | 2 | 168664 | 87014 | 86.75 | 53.39 |
|  |  |  |  | 3 | 159255 | 84314 | 86.98 | 53.57 |
|  |  |  | No | 1 | 138021 | 68717 | 86.83 | 53.16 |
|  |  |  |  | 2 | 121451 | 62467 | 86.59 | 53.23 |
|  |  |  |  | 3 | 92818 | 47710 | 86.83 | 53.85 |

**(B)**
